# Bioactive molecules from terrestrial and seafood resources in hypertension treatment: focus on molecular mechanisms and targeted therapies

**DOI:** 10.1007/s13659-023-00411-1

**Published:** 2023-10-30

**Authors:** Md. Rezaul Islam, Puja Sutro Dhar, Shopnil Akash, Sabeena Hussain Syed, Jeetendra Kumar Gupta, Kumaraswamy Gandla, Muniya Akter, Abdur Rauf, Hassan A. Hemeg, Yasir Anwar, Bassam Oudh Aljohny, Polrat Wilairatana

**Affiliations:** 1 Department of Pharmacy, Faculty of Allied Health Sciences, Daffodil International University, Daffodil Smart City, Birulia, Savar, Dhaka, 1216 Bangladesh; 2 School of Pharmacy, Vishwakarma University, Survey No 2, 3,4, Kondhwa Main Rd, Laxmi Nagar, Betal Nagar, Kondhwa, Pune, Maharashtra 411048 India; 3 Institute of Pharmaceutical Research, GLA University, Mathura, Uttar Pradesh India; 4 Department of Pharmaceutical Analysis, Chaitanya (Deemed to Be University), Himayath Nagar, Hyderabad, Telangana 500075 India; 5 Department of Chemistry, University of Swabi, Anbar, Khyber Pakhtunkhwa, 23561 Pakistan; 6 Department of Medical Laboratory Technology, College of Applied Medical Sciences, Taibah University, Al-Medinah Al-Monawara, Saudi Arabia; 7 Department of Biological Sciences, Faculty of Science, King Abdulaziz University, Jeddah, 21441 Kingdom of Saudi Arabia; 8 Department of Clinical Tropical Medicine, Faculty of Tropical Medicine, Mahidol University, Bangkok, 10400 Thailand

**Keywords:** Antihypertensive, Plants, Bioactive molecules, Seafood, Hypertension

## Abstract

Hypertension (HTN), a complex cardiovascular disease (CVD), significantly impacts global health, prompting a growing interest in complementary and alternative therapeutic approaches. This review article seeks to provide an up-to-date and thorough summary of modern therapeutic techniques for treating HTN, with an emphasis on the molecular mechanisms of action found in substances found in plants, herbs, and seafood. Bioactive molecules have been a significant source of novel therapeutics and are crucial in developing and testing new HTN remedies. Recent advances in science have made it possible to understand the complex molecular mechanisms underlying blood pressure (BP)-regulating effects of these natural substances better. Polyphenols, flavonoids, alkaloids, and peptides are examples of bioactive compounds that have demonstrated promise in influencing several pathways involved in regulating vascular tone, reducing oxidative stress (OS), reducing inflammation, and improving endothelial function. The article explains the vasodilatory, diuretic, and renin–angiotensin–aldosterone system (RAAS) modifying properties of vital plants such as garlic and olive leaf. Phytochemicals from plants are the primary in traditional drug development as models for novel antihypertensive drugs, providing diverse strategies to combat HTN due to their biological actions. The review also discusses the functions of calcium channel blockers originating from natural sources, angiotensin-converting enzyme (ACE) inhibitors, and nitric oxide (NO) donors. Including seafood components in this study demonstrates the increased interest in using bioactive chemicals originating from marine sources to treat HTN. Omega-3 fatty acids, peptides, and minerals obtained from seafood sources have anti-inflammatory, vasodilatory, and antioxidant properties that improve vascular health and control BP. Overall, we discussed the multiple functions of bioactive molecules and seafood components in the treatment of HTN.

## Introduction

**Fig. 1:**
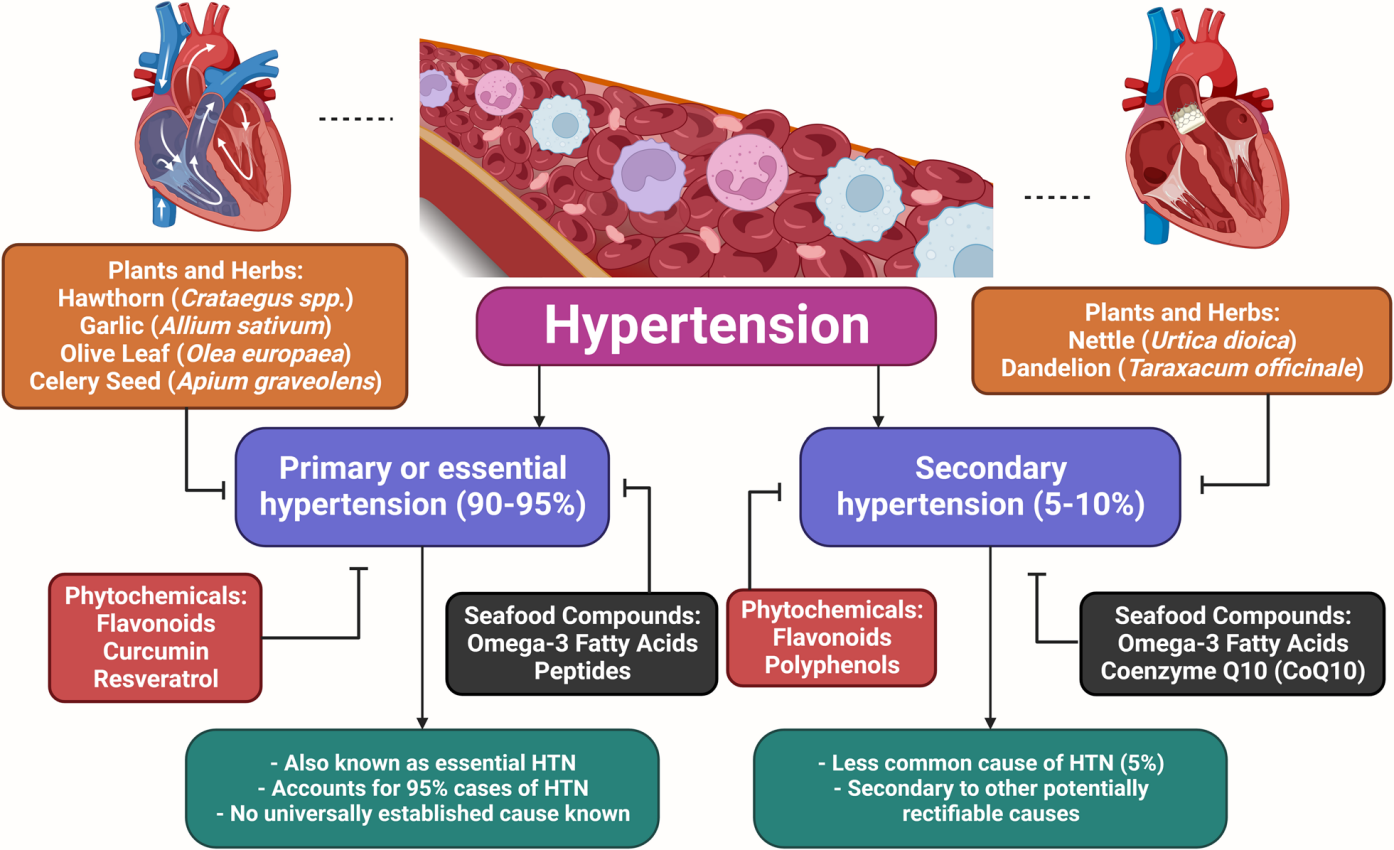
The presentation of the types of HTN and treatment by natural products

Hypertension (HTN), a prevalent chronic disease associated with age, frequently leads to quite significant renal and cardiac issues. Along with blood pressure (BP), a variety of other cardiovascular risk factors are regularly reported [1]. It causes abnormally high BP in the arteries. There are two options: major [2] and secondary (less important) and optional. 90% to 95% of cases of high BP are classified as primary HTN, which means they have no known medical reason [3]. Secondary HTN is brought on by a variety of conditions that affect the arteries, kidneys, endocrine system, or heart in the remaining 4 to 9% of cases [4]. HTN is a major contributor to chronic kidney disease and a risk factor for aneurysms, heart attacks, strokes, and heart failure [5]. A small rise in arterial BP shortens life expectancy. BP can be controlled and the risk of health issues is reduced with the use of medications, dietary changes, and lifestyle adjustments [6]. One of the leading factors in death globally is HTN. Even though a variety of current therapeutics are used to treat clinical HTN, they all have negative consequences. BP is the force that the blood applies to the artery walls. SBP (systolic blood pressure under 120 mmHg) and DBP (diastolic blood pressure under 80 mmHg) are the two classifications. Patients with HTN experience an increase in both SBP and DBP that is greater than 90 mmHg. HTN affects 25.4 percent of the world's population now, with a 60 percent increase anticipated by 2025. HTN is divided into two types (Fig. 1) [7]. Many of today's medicines are obtained directly or indirectly from natural compounds, which have proven to be a great source of innovative medications. This is especially true in cancer and infectious disease research [8, 9]. Meanwhile, natural products have an impact in several circumstances [10]. Around the world, HTN has become one of the top preventable causes of disease and mortality. It is expected to kill 7.5 million people per year, or 12.8 percent of all deaths [11, 12]. The majority of currently used HTN medications cannot be administered as a single-pill therapy due to their low efficacy and side effects. Hence, new medicine development with a wide range of therapeutic effects is highly desirable. Recently advances in the investigation of natural lead compounds with antihypertensive activity, with an emphasis on the mechanisms underlying their antihypertensive effect [13]. Based on molecular mechanisms of action, we reviewed the multiple functions that plants, and seafood components may have in treating HTN. After that, we focused on the phytochemicals and seafood components that are most frequently used to treat and manage HTN. The use of bioactive molecules components in the treatment of HTN has potential. A comprehensive approach is provided by their various molecular modes of action, which include vasodilation, diuretic effects, and the decrease of oxidative stress (OS). For better cardiovascular health, this synergy may result in the successful and balanced control of HTN. Future developments appear promising as we explore deeper into the molecular interactions of bioactive molecules in the treatment of HTN. Individualized treatments focusing on particular processes might become available, improving efficacy and reducing negative consequences. Innovative and comprehensive approaches to managing HTN could be made potential through combined efforts between traditional and modern medications.

## Methodology

A strategy was designed to identify literature published in prestigious journals. All the search results were from PubMed, Scopus, and WOS. The keywords hypertension, bioactive molecules, blood pressure, phytochemicals, and seafood compounds were used. We chose and analyzed original studies, review articles, and research reports published until 2023.

## Causes of HTN

### Essential HTN

"The treatment of the HTN itself is a difficult and nearly desperate task in the current state of our knowledge, and in fact, because knowing the HTN may be a significant compensatory mechanism that should not be tampered with even if it were certain that we could control it." This prevalent misunderstanding concerning the clinical meaning of essential HTN was first articulated by Paul Dudley White in 1931 [14]. The most common type of HTN is essential HTN, which affects 90 to 95 percent of hypertensive persons [3]. Sedentary lifestyles, potassium deficiency (hypokalemia), stress, [15], obesity [16], (those with a body mass index (BMI) higher than 25 account for more than 85 percent of instances) [17], alcohol intake [18], salt (sodium) sensitivity [19], and vitamin D deficiency have all been linked to an increased risk of developing HTN [20]. HTN risk is increased by aging [21], some inherited genetic alterations [22], and having a family history of HTN [23]. An increase in renin, a kidney-secreted enzyme, as well as sympathetic nervous system (SNS) over activity [24], are also risk factors [25]. HTN is hypothesized to be influenced by insulin resistance, which is a part of syndrome X, sometimes known as metabolic syndrome [26]. The rate of preterm birth is lower among children with essential HTN, who also present at an older age, have a greater family history of the condition, and present with essential HTN [27]. The complex character of primary HTN necessitates a comprehensive strategy. Dietary and exercise changes, for example, are essential. Early detection and continuous management are essential due to the interaction between genetic predisposition and environmental factors.

### Secondary HTN

Secondary HTN is high BP that has an underlying, recognizable, and frequently reversible cause. Only 4 to 9 percent of instances of HTN are believed to be the consequence of secondary factors [28]. By addressing the major cause of high BP, this type of HTN differs from essential HTN [29]. HTN can also be influenced by other diseases that cause hormonal imbalances, such as excessive thyroid function, and a lack of thyroid and adrenal gland cancer. Secondary HTN can also be brought on by kidney disease, pre-eclampsia during pregnancy, and coarctation of the aorta [6]. A tiny but considerable portion of the hypertensive population suffers from secondary HTN, which, in contrast to initial HTN, may be treatable [30]. Secondary HTN is caused by recognized underlying conditions such as hormone imbalances [30]. To effectively treat the condition and reduce any potential health risks, it is essential to manage the underlying causes.

## Pathophysiology of HTN

**Fig. 2:**
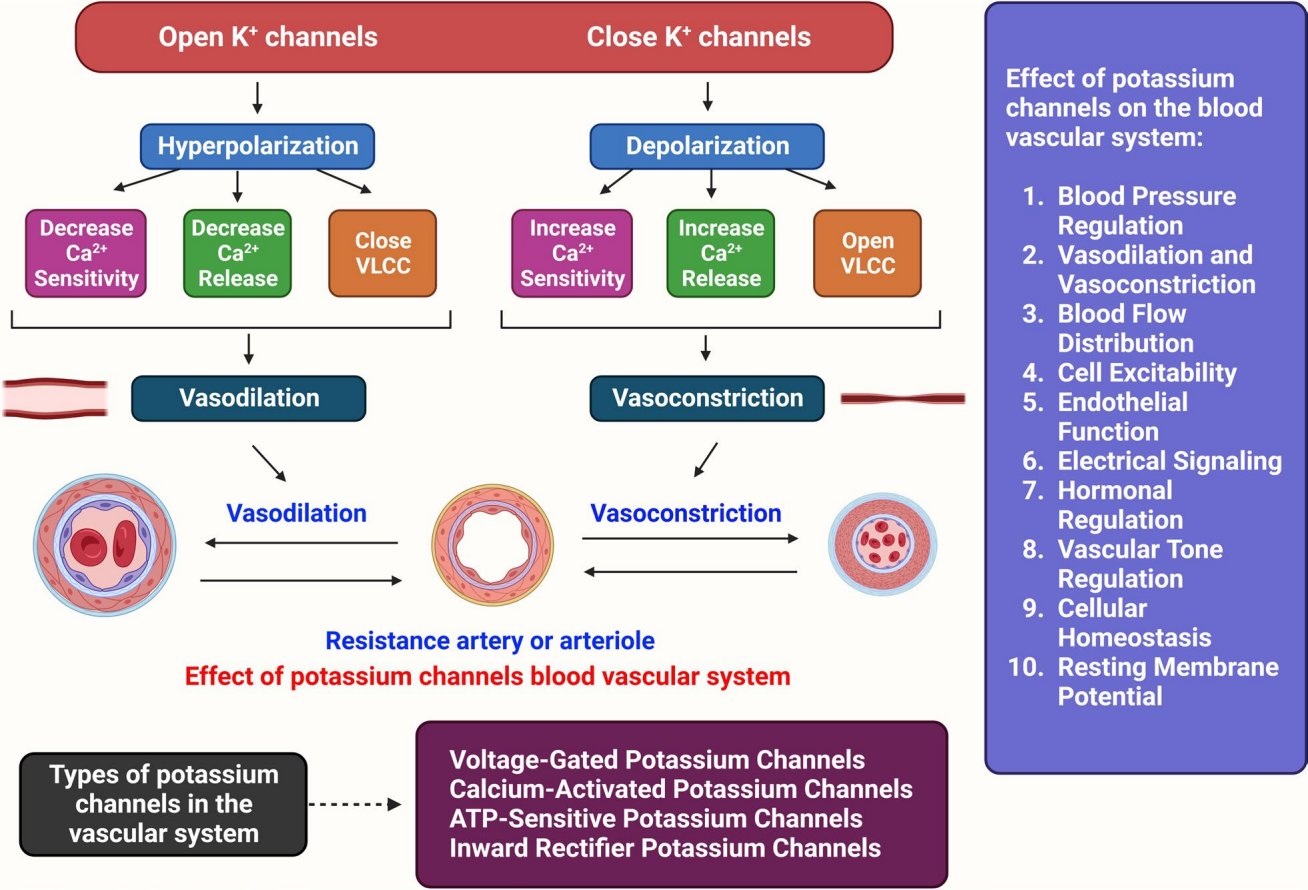
Effect of potassium channels on the blood vascular system

**Fig. 3:**
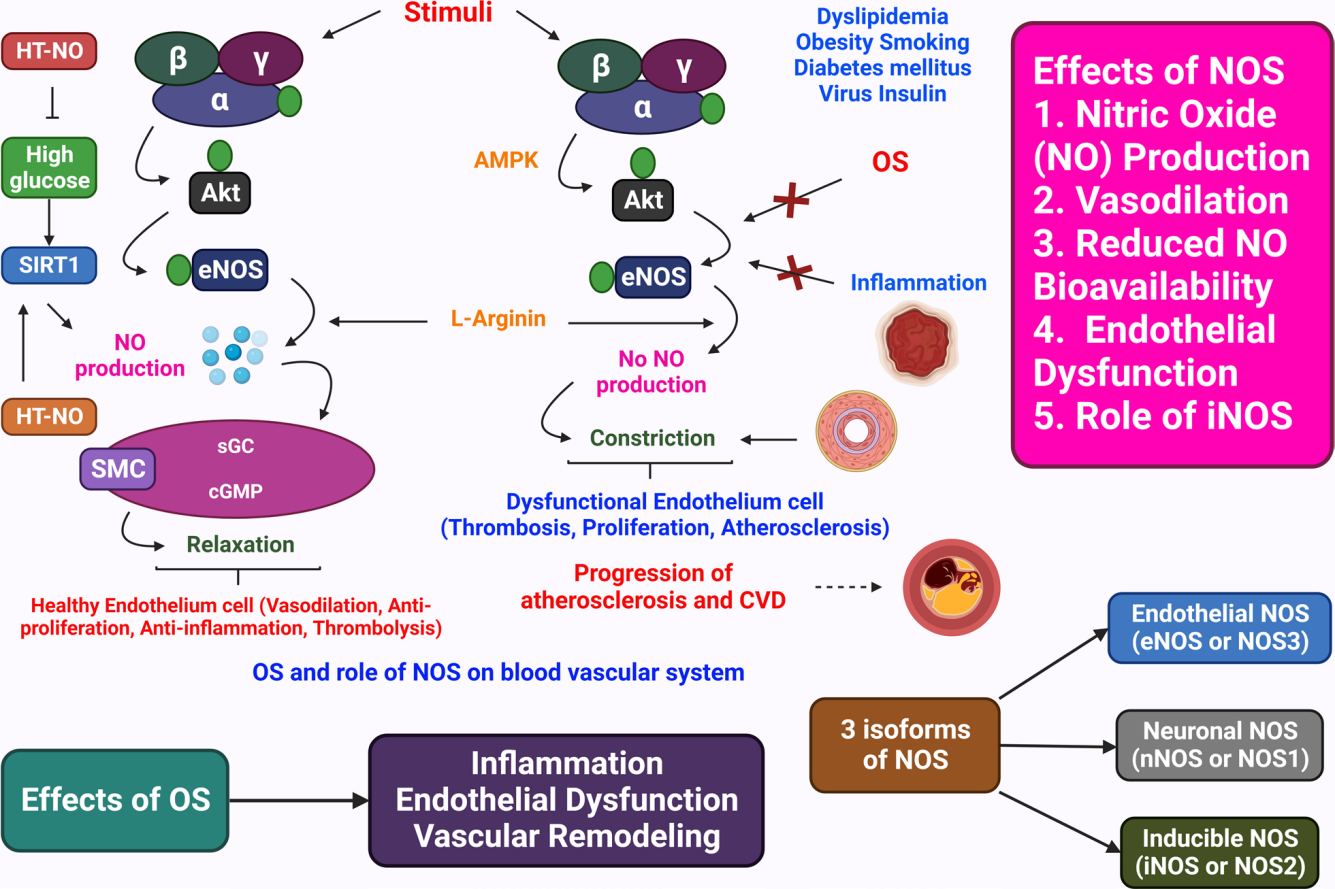
The OS and role of NOS on the blood vascular system

**Fig. 4:**
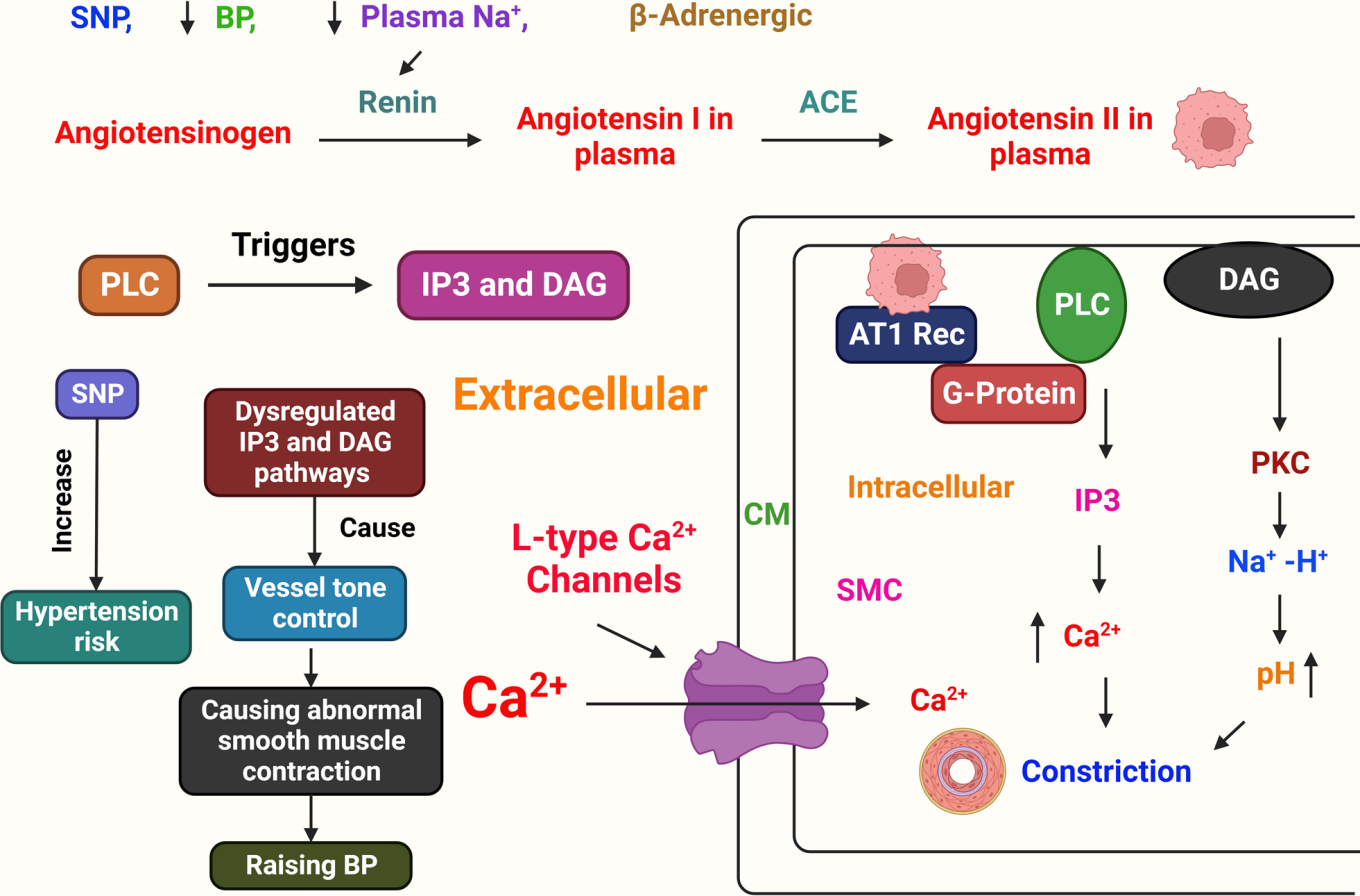
BP management and the function of angiotensin-converting enzyme (ACE). ACE, BP, inositol triphosphate (IP3), diacylglycerol (DAG), single-nucleotide polymorphism (SNP), and phospholipase C (PLC) are examples of these terms

**Fig. 5:**
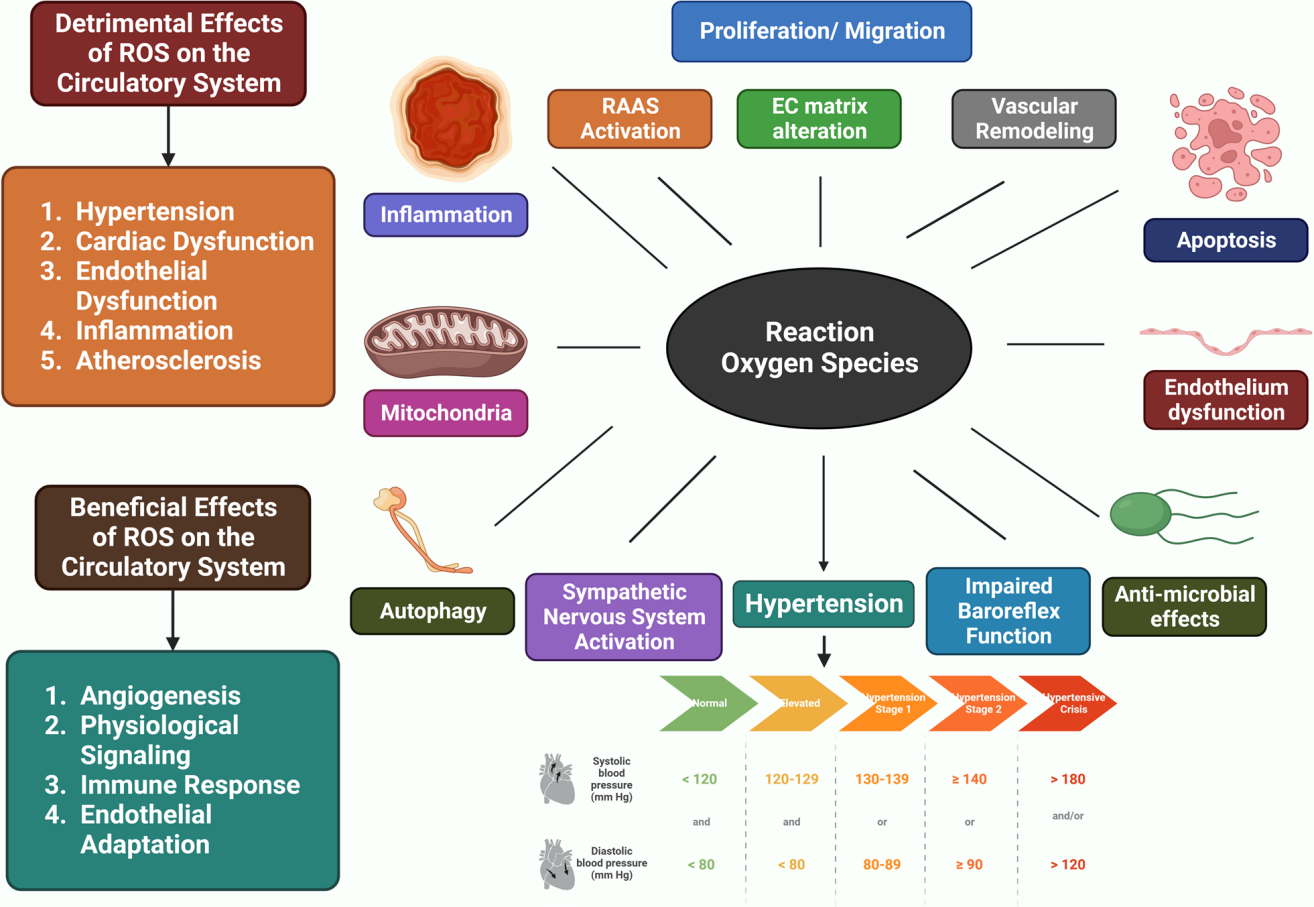
The influence of ROS on the circulatory system

**Fig. 6:**
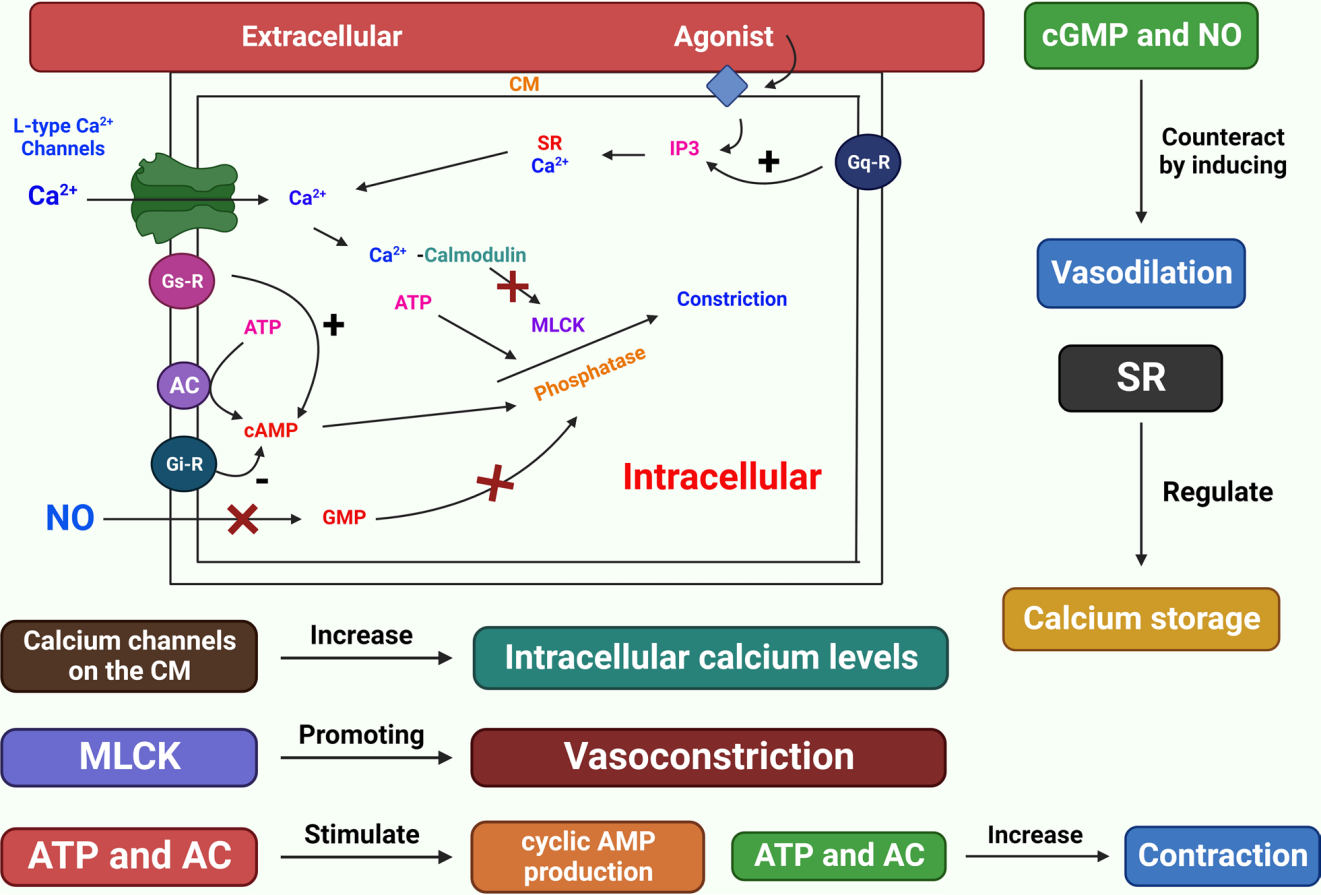
Vasoconstriction caused by calcium channel. Cell membrane (CM), Myosin light-chain kinase (MLCK), ATP, AMP, GMP, NO, adenylate cyclase (AC), and sarcoplasmic reticulum (SR) are all examples of molecules that make up the cell membrane

The etiology of HTN is still unknown . As the underlying cause of their high BP, between 2 and 5% of persons have kidney or adrenal insufficiency [4]. The majority of the processes that cause secondary HTN are known. On the other hand, those related to essential (main) HTN are much less well characterized. The cardiac output gradually recovers to normal as the total peripheral vascular resistance (TPR) increases. To explain this, the three following hypotheses have been put forth: (1) because the kidneys are unable to excrete sodium, natriuretic hormones including atrial natriuretic hormone are released, which speeds up salt excretion and increases TPR. (2) Vasoconstriction, salt and water retention, and hyperactivity of the renin-angiotensin system. HTN results from an increase in blood volume [31]. (3) A SNS that is hyperactive and causes an accelerated stress reaction [32]. (4) Furthermore, HTN is known to be highly genetic and polygenic (caused by numerous genes), and a few potential etiological factors have been proposed [33]. HTN researchers have recently become more interested in the relationship between vital HTN and long-term endothelial damage. However, it is unclear whether endothelial changes occur before HTN or as a result of long-term high BP. High BP is a major contributor to the risk of renal failure, coronary artery disease, and stroke on its own [6]. A lot of elements in the cardiovascular system influence BP, including cardiac output, blood volume, arterial tone balance, and so on. BP is kept in a healthy range by natriuretic peptides, the renin–angiotensin–aldosterone system (RAAS), endothelial cells, the immunological system, and the SNS. Any imbalance in the neurohumoral system's constituent parts might directly or indirectly lead to an increase or decrease in the average BP. A long-lasting imbalance also damages the cardiovascular system in addition to the target organ (such as CKD and left ventricular hypertrophy). Potassium channels [34] (Fig. 2), nitric oxide (NO) (Fig. 3), the renin-angiotensin system (Fig. 4), reactive oxygen species (ROS) (Fig. 5), and calcium ions (Fig. 6) are all physiological effectors that impact vascular tone, and any imbalance in these variables can lead to HTN. The bioavailability of NO is also decreased by superoxide, and superoxide generation is increased through uncoupled endothelial nitric oxide synthase (eNOS), which increases OS, a primary contributor to HTN [35]. Narrowed arteries, higher cardiac output, and inadequate kidney function all contribute to the pathophysiology of HTN, which results in raised BP. Targeted therapy and risk reduction are made easier with an understanding of these mechanisms.

As shown in (Tables 1 and 2), nature has developed or inspired a variety of lead compounds that can successfully alter these features. The information in the section that follows relates to popular traditional herbs that might have antihypertensive properties.

**Table 1:** Medicinal plants and natural compounds have antihypertensive properties

Medicinal herbs	Experimental model, dose, and targets	Mechanism of action	Types of study	Activity and molecular mechanism	Major findings	References.
*Allium sativum*	Rats that were fed fructose,	Blocks ACE	In vivo	Vasorelaxant	Block the migration and development of cells triggered by angiotensin II	[34]
	Rat's isolated pulmonary arteries, 100 mM, Angiotensin II-stimulated cell-cycle progression,	Enhance NO	In vivo	Vasorelaxant, AMS, and DAS inhibit cell-cycle progression and migration by preventing p27 downregulation and reducing ERK1/2 phosphorylation	Potential for inhibiting the enzymes cyclooxygenase (CO), 5-lipoxygenase (LO), ACE, and thrombocyte aggregation	[36]
	Human umbilical vein Endothelial cells have inhibitory properties on various enzymes, including 5-lipoxygenase, cyclooxygenase, thrombocyte aggregation, and angiotensin I-converting enzyme	Enhance NO	In vivo	Vasorelaxant, the sources provided did not explicitly mention the molecular mechanisms of action for the inhibitory effects on 5-LO, CO, TA, and ACE	In rat heart tissue, endogenous antioxidants are induced dose-dependently	[37]
	Aortic rings from Sprague–Dawley rats, 125, 250, 500, 1000, and 2000 mg/kg	Enhance H_2_S	In vivo	Vasorelaxant, the process by which garlic increases endogenous antioxidants at lower dosages	Drugs for the Prevention and Treatment of CVD	[38]
	Rats that were fed fructose, 150 mg/kg/day and 400 mg/kg/day, the target subjects in the study were fructose-fed rats	Lowers the NADPH activity	In vivo	Antioxidant, Rats fed fructose experienced decreased OS, increased eNOS activity, and reduced expression of VCAM-1	eNOS activity was raised and OS was reduced	[39]
	Neutrophils in humans, 0.80 mg/ml, AGE significantly inhibits superoxide production	Scavenges ROS	–	Antioxidant, AGE exhibits a 54% inhibitory action on superoxide radicals in the HPX-XOD system and 37% in the viable cell system	Avoiding ROS	[40]
	Rats fed a high fructose diet, 250 mg/kg/day, the intervention's focus was on the cardiac complications in insulin-resistant diabetic rats	NF-κB inhibition	In vivo	Anti-inflammatory increased cardiac H_2_S levels, PI_3_K/Akt pathway activation, and lower keep levels in the fructose-fed heart following garlic administration may be the causes of this rise in Nrf2 levels	Reducing myocardial OS and cardiac enlargement caused by fructose	[41]
*Apium graveolens*	Aortic rings were isolated from rats; the target of the research work was to determine whether celery extracts may reduce OS in mice that had received CCl_4_ treatment	Ca^2+^ channels inhibitions	In vivo	Vasorelaxant, as evidence of their antioxidant action, extracts were discovered to be effective free radical scavengers (OH• and DPPH•) and to decrease the level of liposomal peroxidation	The effects of OS in mice	[42]
	Mice exposed to CCl_4_, 5, 10, and 20 mg/kg body weight, D-carvone was used to treat rat HTN brought on by Nω-nitro-l-arginine methyl ester hydrochloride (l-NAME)	Increase antioxidants	In vivo	Antioxidant, the agent that lowers BP, lowers cholesterol and acts as an antioxidant when l-NAME causes HTN	Impact of antioxidants	[43]
*Camellia sinensis*	Brachial arteries of people with coronary heart disease, 450 mL of short-term black tea and 900 mL of long-term black tea daily for 4 weeks, Effects of black tea consumption on patients with coronary artery disease’s endothelial dysfunction	Causes flow-mediated dilation (FMD) to increase	In vivo	Vasorelaxant, Consuming tea enhanced endothelial function while only slightly raising SBP	Tea consumption and lowered CVD	[44]
	Brachial arteries of people with coronary heart disease, 5 cups per day of black tea for 4 weeks, regular black tea consumption can enhance brachial artery vasodilator activity	Causes flow-mediated dilation (FMD) to increase	–	Vasorelaxant, an increase in the availability and/or activity of NO may serve as a partial mediator for these pathways	Black tea may reduce CVD by increasing the conduit artery vasodilator effect	[45]
	Superoxide-producing apparatus, NO and superoxide were directly scavenged by green tea extract and green tea tannin combination in vitro	ROS Scavenging	In vitro	Antioxidant, direct scavenging of NO and O2A by green tea tannins suggests that ONOO A production has been inhibited	Green tea tannin contains antioxidant properties	[46]
	In endothelial cells grown in culture, EGCG also inhibits the IL-1β-induced induction of VCAM-1 expression	Decrease VCAM-1	In vitro	Anti-inflammatory, EGCG decreases the TNF-α-induced adhesion of THP-1 cells to HUVECs	A flavonoid (12) contained in tea called epigallocatechin-3-gallate reduces VCAM-1 expression brought on by cytokines	[47]
	Endothelial cells from humans reduced the risk of mortality from CVD	NF-κB inhibition	In vivo	Anti-inflammatory	CVD decreased	[48]
	STZ fed SHR, 13.3 g/L in tap water for 12 weeks, in diabetic hypertensive rats, the goal of GT therapy was to reduce kidney damage	NADPH oxidase reduces	In vivo	Antioxidant, in diabetic hypertensive rats, it was found that GT reduced the expression of Nox4, a significant source of formation of ROS in the kidney, lowering OS and renal injury	In people with diabetes and HTN, lessened nephropathy	[49]
	Strong man smokers (preclinical pilot), 2 g/kg of diet and 4 g/kg of diet	Enhance NO	In vivo	Vasorelaxant	Impact on insulin resistance and the lipid profile	[50]
	For diabetic SHR, the target of GT treatment is to enhance NO bioavailability and decrease OS	eNOS uncoupling inhibition	–	Vasorelaxant, NO bioavailability reduces and eNOS is uncoupled when tetrahydrobiopterin (BH_4_) levels are low in diabetes circumstances. By reversing the decrease in BH_4_ levels and raising the ratio of dimers to monomers of eNOS, GT therapy reduced this	Reduced endothelial NO synthase uncoupling	[51]
	C57BL/6 mice, 12 weeks and exposed to 5 μmol PCB 126/kg mouse weight (1.63 mg/kg-day) on weeks 10, 11, and 12, with a total body burden of 4.9 mg/kg	Increase antioxidants	In vivo	Antioxidant, Nuclear factor (erythroid-derived 2)-like 2 (Nrf2) and aryl hydrocarbon receptor (AhR) proteins regulate gene transcription, which in turn mediates the antioxidant response	Limit OS caused by PCB 126	[52]
	Human smooth muscle cells in the aorta, 381.17 μg/mL	Enhance HO-1 enzyme	In vivo	Anti-proliferative	Impact on BP	[53]
*Coptis chinensis*	Aortic endothelial cells from rats, in the context of endothelial dysfunction, adenosine monophosphate-activated protein kinase (AMPK) is the molecular target of berberine	NF-κB reduces	In vivo	Anti-inflammatory, eNOS is phosphorylated at Ser1177 by berberine, which causes eNOS to associate with heat shock protein 90 (HSP90) and activate AMPK	Vasodilatation enhanced	[54]
	Rat cardiomyocytes (hypertrophy induced by insulin)	Increases the expression of eNOS	In vivo	Vasorelaxant, the ATP-sensitive K^+^ channels may open, the concentration of NO may rise, the sarcoplasmic reticulum's function to release Ca^2+^ is inhibited, and the cell membrane may allow external Ca^2+^ to enter	Impact on vasoconstrictive activity	[55]
	Thoracic aortic rings in CIHH rats	Increases the expression of eNOS	In vivo	Vasorelaxant	Having an impact on vasoconstrictor action	[55]
	Atherosclerotic renovascular disease (ARD) in Wistar rats	Amplify antioxidants	In vivo	Antioxidant, Berberine decreases the expression of phosphorylated IKKb, NF-κB subunit p65p50, pro-inflammatory protein iNOS, and profibrotic factor TGF-b and also down-regulates NF-κB activity	Minimizing atherosclerotic renovascular disease-induced chronic kidney damage	[56]
*Coriandrum sativum*	Wistar albino rats' hepatotoxicity caused by CCl_4_, 100 and 200 mg/kg body weight	Enhances antioxidants	In vivo	Antioxidant, the extract increased the activities of antioxidant enzymes like SOD, CAT, and GPx while significantly reducing the levels of thiobarbituric acid reactive substances (TBARS) and serum marker enzymes caused by CCl_4_	Effects that prevent the hepatotoxicity that carbon tetrachloride causes	[57]
	Reduces the NF-κB, the anti-inflammatory properties of *Coriandrum sativum* aerial components (stem and leaf) on lipopolysaccharide (LPS)-stimulated RAW 264.7 macrophages	LPS-stimulated RAW 264.7	–	Anti-inflammatory, NO, prostaglandin E2 (PGE2), iNOS, cyclooxygenase-2 (COX-2), and pro-interleukin-1 (IL-1) production were all inhibited by *Coriandrum sativum*	Reduced inflammatory reactions brought on by LPS	[58]
*Crataegus *spp.	Rats with HTN brought on by l-NAME, 100 mg/kg for 4 weeks, to prevent l-NAME-induced HTN in rats	eNOS is activated	In vivo	Vasorelaxant, had a hypotensive effect and prevented l-NAME-induced HTN in rats	Avoid l-NAME-related HTN	[59]
	An enzyme assay, protecting DNA from OS-related damage was the target of ECPP. In mouse lymphocytes, it also demonstrated a cytoprotective effect against H_2_O_2_-induced DNA damage	ROS scavenging	–	Antioxidant, in mouse lymphocytes, it has a cytoprotective effect against H_2_O_2_-induced DNA damage	DNA damage response to OS: the protective effects of extract	[60]
	STZ-produced diabetes in rats, 100 mg/kg for 4 weeks	Lowers TNF-α	In vivo	Anti-inflammatory, extract included the prevention of lipid peroxidation, reduction in plasma levels of TNF-α, interleukin-6 (IL-6), and the reduction of inducible iNOS expression in the aorta	Implications for vascular dysfunction	[61]
*Crocus sativus*	Using genotoxins on Swiss albino mice, 20, 40, and 80 mg/kg body weight for 5 days	Increased antioxidants	In vivo	Antioxidant, saffron extract increased the activity of the phase II enzyme GST, indicating the fast removal of genotoxic metabolites or free radicals produced by the genotoxins	Protective impact on OS brought on by genotoxins	[62]
	Heart from a single guinea pig, 50, 100, and 150 mg/kg	Ca^2+^ channels blocks	In vivo	Vasorelaxant	Using antihypertensive medication to combat induced HTN	[63]
	Wistar rats treated with BeCl_2_, 86 mg/kg b.w. orally for 5 days, study crocin's ability to protect rats from BeCl_2_ intoxication	Decreased OS	In vivo	Antioxidant, by lowering OS and increasing the gene expression of antioxidant enzymes, crocin has been found to protect against the toxicity of BeCl_2_. By scavenging free radicals and inhibiting lipid peroxidation, it protected DNA from oxidative damage	OS decreased	[64]
	Ischemia–reperfusion-treated rats, 0.1–0.5 mL/kg/day, for 14 days, through the phosphorylation of Akt/GSK-3/eNOS and the reduction of IKK-β/NF-κB protein expressions in the myocardium, safranal demonstrated its protective effects against IR injury	eNOS is activated	In vivo	Vasorelaxant, by increasing Bcl-2 expression, lowering Bax and caspase3 expression, and reducing TUNEL positivity, safranal demonstrated anti-apoptotic potential	Akt/GSK-3/eNOS phosphorylation regulates the protection that safranal causes in the heart	[65]
*Panax*	For rat cardiomyocytes with oxidative damage after hypoxia/reoxygenation, the ginseng treatment target seeks to decrease the condition and decrease OS	Enhances antioxidants	In vivo	Antioxidant	OS should be decreased to prevent chronic cyclosporine nephropathy	[66]
	Macrophages from mice, the innate immunological response in macrophages is the target of ginsenoside Rg1	NF-κB decreased	In vivo	Anti-inflammatory, Ginsenoside Rg1 controls the innate immune response in macrophages by influencing the PI3K/Akt/mTOR and NF-κB pathways differently. It reduces NF-κB and I-B activation, which results in decreased mRNA levels of pro-inflammatory cytokines	Differentially modulate the NF-κB and PI3K/Akt/mTOR pathways to regulate macrophage innate immunological responses	[67]
*Salviae miltiorrhizae*	CHD sufferer, 5 g extract, twice per day for 60 days, to decrease OS	Enhances antioxidants	In vivo	Antioxidant, in comparison to the placebo group, SMHE significantly decreased the levels of malondialdehyde (MDA), increased levels of GSH, and boosted the activities of SOD, paraoxonase (PONase), and glutathione reductase (GSSG-R)	The impact on antioxidant enzymes	[68]
	Thoracic aortic vascular smooth muscle cells (VSMCs) of Sprague–Dawley rat, Trastuzumab, a monoclonal antibody that targets erbB2 receptors	Decrease ROS	In vivo	Antioxidant, Trastuzumab treatment inhibits tumor growth by targeting the neuregulin-1/ErbB signaling pathway, which increases breast cancer patients' metastatic potential by overexpressing erbB2 receptors	Neuregulin-1/ErbB signaling's effects on cardio protection involve vascular signaling and angiogenesis	[69]
*Nigella sativa*	Aorta isolated by SHR, when macrophages are activated by lipopolysaccharide (LPS), luteolin suppresses the release of proinflammatory cytokines with a half-maximal inhibitory concentration (IC_50_) of between 1 and 5 microns	Increase Na^+^, K^+^, and Cl^−^ in urine	In vivo	By reducing the activity of a luciferase reporter gene in macrophages that have been transfected with it and are under the control of NF-κB cis-acting elements, luteolin can show that it inhibits NF-κB-mediated gene expression	Effects that are diuretic and hypotensive	[70]
*Cymbopogon citratus*	Thoracic aorta in a rat	Ca^2+^ influx blocks	In vivo	Vasorelaxant	Impact on CVD	[71]
	Isolated aorta from WKR, 1–20 mg/kg	NO bioavailability increases	In vivo	Vasorelaxant, citronellol inhibits the contractions caused by calcium chloride (CaCl_2_) and blocks the contractions induced by phenylephrine or caffeine	Having vasorelaxant and hypotensive effects	[72]
*Bidens pilosa* L	Rats fed a high fructose diet, In adipocytes and animals, *Bidens pilosa* and its active ingredient, 2-β-D-glucopyranosyloxy-1-hydroxytrideca-5,7,9,11-tetrayne (GHT), target adipogenesis and lipid accumulation	Mechanism not determined	In vivo	Vasorelaxant, Peroxisome proliferator-activated receptor (PPAR), CCAAT/enhancer binding proteins (C/EBPs), and Egr2 are all decreased by *Bidens pilosa* and GHT in adipocytes, which suppresses adipogenesis and lipid content	Suppress lipid aggregation and adipogenesis	[73]
	RAW 264.7 after LPS stimulation, in MCF-7 cells that had been treated with 12-O-tetradecanoylphorbol-13-acetate (TPA) inhibited the transcription of cox-2	Activation of TNF-α	In vitro*, *In vivo	Anti-inflammatory, by preventing NF-κB from binding to its cis-acting element, ethanol inhibited NF-κB activation and reduced the production of iNOS and COX-2	Inhibits NF‐κB activation	[74]
*Andrographis paniculata*	SHR, in HL-cells that have differentiated into neutrophils, andrographolide (02) has been shown to prevent the activation of NF-κB caused on by PAF and *N*-formyl-methionyl-leucyl-phenylalanine (fMLP)	ROS Scavenging	–	Antioxidant, the DNA binding of NF-κB in whole cells and nuclear extracts induced by PAF and fMLP has been found to be decreased by andrographolide (02)	Impact on CVD	[75]
	Hearts from Sprague–Dawley rats	NO enhances	In vivo	Vasorelaxant, reduced levels of free radicals in the kidneys and plasma-circulating ACE	Hypotensive activity	[76]
	Gene-knockout mice for Npr1, IKK activity reduction has been proposed as a therapeutic approach target	Inhibits NF-κB	In vivo	Anti-inflammatory, IKK is a main component in NF-κB activation, and IB is essential for controlling NF-κB activity	The Npr1 gene has anti-regulatory effects on NF‐κB signaling, renoprotective effects, and HTN prevention	[77]
*Zingiber officinale*	An enzyme assay, for the purpose of cellular defense protection and prevention of diseases connected to ONOO, Zingerone has been proposed as a potential ONOO scavenger	ROS scavenging	–	Antioxidant, electron donation is the molecular basis of zingerone's ability to scavenge, and it prevents tyrosine from being nitrated and ONOO– from forming	Impact of antioxidants	[78]
	Heart of rat, depending on the particular condition being targeted, different amounts of ginger may be required for different bioactivities and health advantages	lipid peroxidation blocks	In vivo	Antioxidant, inhibiting pro-inflammatory molecules and signaling pathways may be how ginger also has anti-inflammatory benefits	Impact of antioxidants	[79]

**Table 2:** Medicinal plants, herbs, and natural compounds have antihypertensive activity

Herbs	Dose/duration, and target	Types of study	Condition and molecular mechanism	Design/population size	Result/magnitude of result	Major findings	References.
*Allium sativum*	2500 mg of garlic daily for ten days, the myocardial, or heart tissue, is the primary target of garlic's effects on endogenous antioxidants in the rat heart	In vivo	Mild HTN, Low concentrations of allicin (01) and its metabolites can cause minimal OS without endangering cells, while higher concentrations may lead to overactive and cardiotoxic effects	Crossover, controlled by placebo/7	Decreased SBP/16 mmHg	When OS causes harm, the heart can benefit from cytoprotective measures	[38]
	950 mg AGE every day for 12 weeks, the BP of patients with treated but uncontrolled HTN is the target of interest	In vivo	Uncontrolled HTN, Garlic's ability to lower BP is thought to be due to its ability to increase intracellular synthesis of NO, H_2_S, and angiotensin II	Randomized, parallel, placebo-controlled, double-blind study/49	Decreased SBP/11.2 ± 4.3 mmHg	SBP reduced	[80]
	470 mg AGE every day for 12 weeks, to decrease BP in patients with uncontrolled HTN	In vivo	Uncontrolled HTN, effects of BP	Randomized, parallel, placebo-controlled, double-blind study/78	Decreased SBP/12.8 ± 5.4	Treatment for uncontrolled HTN that works	[81]
	4 weeks of taking 400–1600 mg/day of powdered garlic, decrease in both SBP and DBP	In vivo	Stage 1 HTN, effects of BP	Placebo-controlled, randomized, parallel/211	Decreased DBP and SBP/9.3 and 6.25 mmHg	Impact on SBP and DBP	[82]
*Camellia sinensis*	40 cc of water and 7.6 g of tea leaves heated for 1 h	In vivo	Mild HTN, effects of BP	Placebo-controlled, double-blind,/19	Increased SBP and DBP/1.6 and 0.8 mmHg (green tea) 0.6 mmHg each (black tea)	Impact on BP	[83]
	378 mg of extorted green tea per twelve weeks, the impact of green tea extract (GTE) on these patients' cardiovascular risk factors, including insulin resistance	In vivo	Obese, HTN, the active ingredient in GTE, EGCG, has been demonstrated to have antioxidant, anti-inflammatory, and anti-atherosclerotic actions	Placebo-controlled parallel, randomized/55	Decreased DBP and SBP/5 each mmHg	Inflammatory biomarkers, BP, and OS reduced	[84]
	3 cups of black tea per day at 1492 mg each for 24 weeks equals 4478, BP-lowering effects	In vivo	Mild HTN, tea flavonoids (12) can enhance NO levels and improve endothelial function, which could decrease vascular tone and reduce BP	Placebo-controlled parallel, randomized,/94	SBP and DBP reduce /3 and 2.0 mmHg	Impact on BP	[85]
*Crataegus* spp.	500 mg extort each day for 10 weeks, the phosphorylation of Akt and eNOS is induced by the extract	In vivo	Mild HTN, the extract activates eNOS through a redox-sensitive Src-PI3-kinase-Akt pathway	Placebo-controlled parallel, randomized, double-blind /35	DBP/12.1 mmHg	WS® 1442 transported NO-mediated relaxations of coronary artery rings that were endothelium-dependent	[86]
	Flavonoids (12) (Hydro-alcoholic extract) at 2.6–4 mg/day for 4 months, to study saffron's hypotensive effects	In vivo	Mild HTN, hypotensive effects	Placebo-controlled parallel, randomized, double-blind /91	Decreased SBP and DBP/12 and 9 mmHg	Effect on hypotension	[87]
*Crocus sativus*	400 mg per day for seven days, the extract has a strong inhibitory effect on the guinea pig heart's calcium channel	In vivo	Healthy, the extract's ability to inhibit calcium channel function in the isolated guinea pig heart suggests its potential as a calcium channel blocker	Placebo-controlled parallel, randomized, double-blind /29	Decreased MAP and SBP/12 and 4 mmHg	Calcium channel inhibition	[88]
*Nigella sativa*	200 and 400 mg/day of seed extract in water (100 and 200 twice a day) for 8 weeks, to evaluate the antihypertensive effect	In vivo	Mild HTN, the extract's ability to lower SBP and DBP in a dose-dependent way	Placebo-controlled randomized, double-blind/107	Decreased SBP and DBP /2.3 & 1.2 mmHg LDL-cholesterol reduction	BP decreased	[89]
*Panax*	*Panax quinquefolius* 3 g/day for 12 weeks, to inhibit leukocyte chemotaxis and possess anti-inflammatory properties	In vivo	Essential HTN, Tanshinone may suppress the synthesis of inflammatory cell cytokines and the metabolism of arachidonic acid as part of its anti-inflammatory action	Placebo-controlled randomized, double blind/63	SBP reduce/16.4 mmHg	Impact on BP	[90]
	300 mg/day of *Panax ginseng* extract for 8 weeks	In vivo	Mild HTN, Danshen, and Gegen therapy prevent atherosclerosis in high-risk hypertensive patients by increasing flow-mediated dilation (FMD) and reducing carotid intima-media thickness (IMT)	Placebo-controlled randomized/91	Reduced SBP and DBP/3.2 and 2.4 mmHg	Benefits of preventing CVD in high-risk HTN	[91]
	400 mg per 3 h, to assess the acute effects of Korean red ginseng (Rg3-KRG) ginsenoside Rg3-enriched on BP	In vivo	Healthy, the molecular mechanism of Rg3-KRG is believed to be the modulation of vascular function	Crossover, randomized, double blind /22	Reduced SBP and DBP /5.8 and 3.5 mmHg	Reduces both central and peripheral arterial pressures is Rg3-KRG extract	[92]

## Medicinal plants and their bioactive compounds having hypotensive/antihypertensive effects

**Fig. 7:**
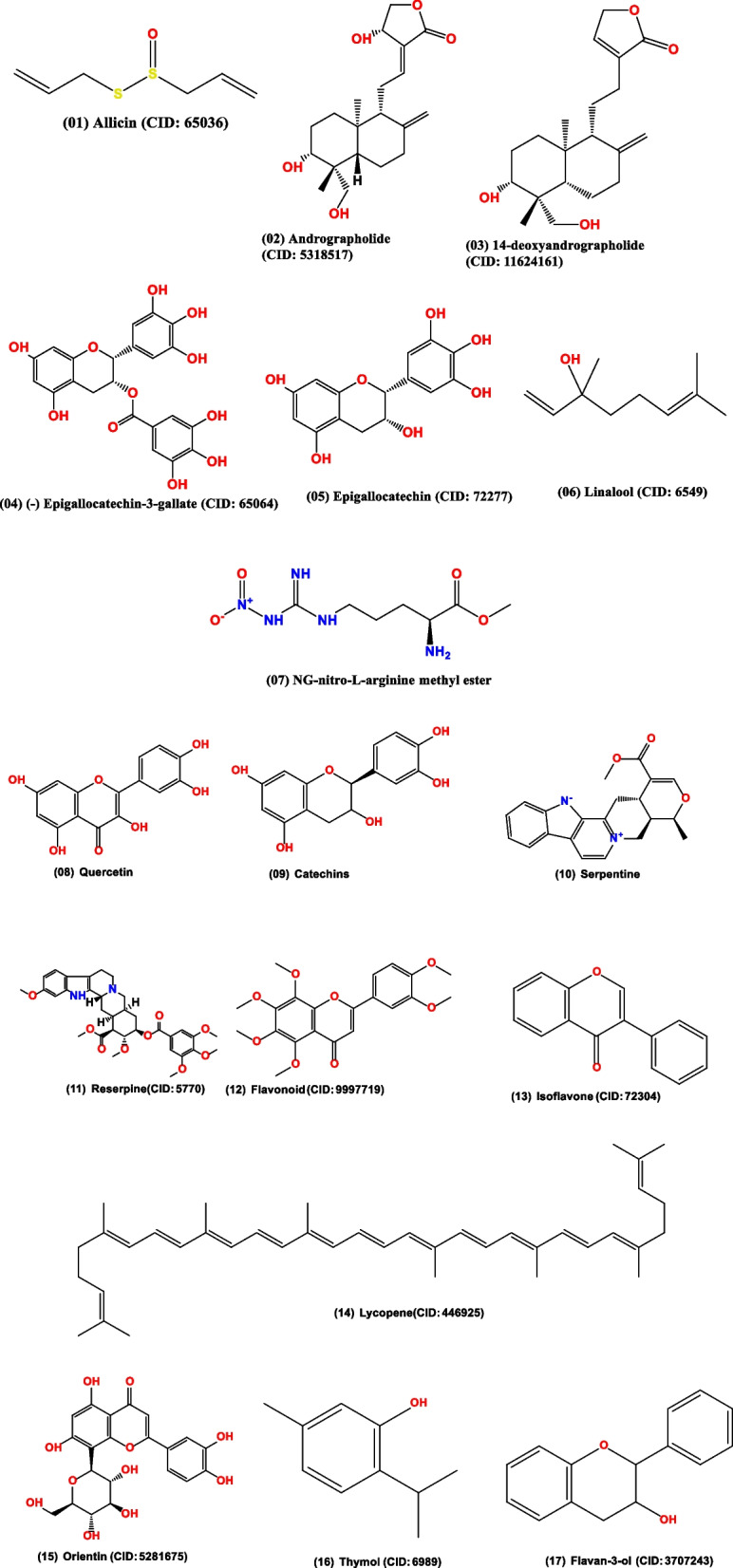
The chemical structures of natural compounds. These compounds have antihypertensive activity

The medicinal plants, bioactive compounds used to treat HTN because of have antihypertensive activity (Fig. 7). Garlic, hibiscus, and omega-3 fatty acids are just a few examples of medicinal plants, herbs, and substances that have hypotensive properties. Utilizing these natural therapies can enhance existing medical procedures and improve cardiovascular health.

### *Allium sativum*

#### *Allium sativum*: (Family: Alliaceae; Common name: Garlic)

Garlic known as *Allium sativum* is one of the first recognized medicinal plants, and it has been used for many years to cure a range of human diseases [93]. For many years, garlic has been used to treat many heart diseases, particularly hyperlipidemia. According to mythology, it possesses hypotensive properties as well. NO is thought to relax smooth muscles and dilate blood vessels when it is produced more frequently. The major chemical constituents of garlic is allicin (**01**) that provides its distinctive smell and many of its health benefits [94]. Formulations including garlic were observed to lower BP in HTN patients more effectively than a placebo [94]. Lower BP, a considerable reduction in lipid peroxidation, NO, and 8-hydroxy-2-deoxyguanosin, as well as an elevation in antioxidant vitamins, were all found (C and E). In this investigation, garlic was found to have a positive cardioprotective effect in essential HTN [95]. Alliums should only be used sparingly since they inhibit thiol group enzymes, which are inhibited by allyl and related sulfoxides [96]. Garlic has antioxidant characteristics that make it a promising treatment for HTN. Garlic consumption could be a healthy and supportive method of controlling BP.

### *Apium graveolens*

#### *Apium graveolens*: (Family: Apiaceae; Common name: Celery)

The apiaceae family includes the annual or perennial plant *Apium graveolens* L., which grows in tropical and subtropical regions of Africa, Asia, and Europe [97]. Because celery affects the liver, which is associated to one type of HTN in Chinese belief, it is helpful for HTN [98]. *Apium graveolens* seeds can be utilized as a secure and efficient treatment for high BP, as shown by the significantly different BP readings in humans before and after treatment [99]. By combining fresh celery juice with vinegar, HTN-related headaches, shoulder pain, and dizziness can be relieved. Furthermore, it is used to treat climacteric HTN and HTN associated with pregnancy [100]. Plants are used to cure diseases because of their composition and therapeutic characteristics, which requires ongoing study into other beneficial and undiscovered aspects of them [97]. *Apium graveolens* used to decrease BP by relaxing blood vessels.

### *Coleus forskohlii*

#### *Coleus forskohlii*: (Family: Lamiaceae; Common name: Karpurvali)

For a variety of conditions, *Coleus forskohlii* is frequently used internationally. The leaves have emmenagogue, diuretic, and expectorant properties in Egypt and Africa. In Brazil, it is used to treat intestinal issues and as a stomach benefit [101]. On isolated rabbit and cat hearts in vivo, it has a favorable inotropic effect at low dosages. Although it does not affect the guinea pigs' bronchial musculature, coleonol has a spasmolytic impact on the gastrointestinal smooth muscle in many other species. Coleonol has a depressant effect on the central nervous system when taken in large dosages [102]. In addition, coleonol has non-specific spasmolytic effects on the smooth muscles of the gastrointestinal tract in a variety of species, but not on the guinea pig's bronchial musculature [102]. *Coleus forskohlii* can be used to dilate blood vessels and improve heart function, perhaps assisting in the treatment of HTN. Combining it with medical advice can be advantageous.

### *Crataegus pinnatifida*

#### *Crataegus pinnatifida*: (Family: Rosaceae; Common name: Chinese Hawthorn)

The Chinese hawkhorn known as *Crataegus pinnatifida* is readily available and has a long history of usage in traditional medicine [2]. It has been proven in pharmacological and clinical tests to reduce BP. The two main antioxidant compounds found in hawthorn, flavonoids (12), and oligomeric procyanidins, are what primarily contribute to the fruit's positive benefits on the heart. By lowering BP, boosting circulation, and preventing plaque buildup on arterial walls as well as blood clot formation in the brain, heart, and arteries. The alkaloid Rhynchophylline which is found in cat's claw, may help to prevent strokes and heart attacks [103]. Intravenous administration of the extract preparation decreased BP in rabbits that had been anesthetized for up to 3 h [104]. It was discovered that the principle causing hypotension. The medication of *Crataegus pinnatifida* has a broad impact on the cardiovascular system. One of the negative effects of nitrous oxide stimulation is hypotensive activity via vasorelaxation [105]. It exerts antioxidant and tonifying effects on cardiac myocytes [106]. The majority of experimental findings support the fact that *Crataegus pinnatifida* has potent pharmacological effects when used alone, but given current understandings of disease pathophysiology [2]. Hawthorn has advantages for the heart, including lowering BP. Along with medical counsel, including hawthorn into one's lifestyle may help with a complete approach to managing HTN.

### *Daucus carota*

#### *Daucus carota*: (Family: Umbelliferae; Common name: Carrot)

The most significant root vegetable plant farmed worldwide is the edible carrot, *Daucus carota*, which primarily consists of two parts: the stem and the root. The majority of the root is made up of periderm, which has an inner core and an outside cortex that is pulpy [107]. It has been used to treat HTN in conventional medicine. According to in vitro tests, these substances reduced arterial BP in rats under anesthesia in a dose-dependent manner (1–10 mg/kg). Both substances showed a dose-dependent (10–200 g/mL) inhibitory impact on rabbit aorta contractions caused by K^+^ and spontaneously beating guinea pig atria at comparable doses [108]. Daucusol and daucuside, two new guaiane-type sesquiterpene terpenoids with an exciting epoxy unit, were found in the fruits of *Daucus carota* [109]. The investigations on carrot pomace stated above lead to the conclusion that more healthy products could be developed by utilizing carrots' functional, nutritional, and medicinal properties [110]. Carrots have nutrients that may help with BP control. While not a cure-all, including carrots in a balanced diet may improve cardiovascular health in general.

### *Desmodium styracifolium*

#### *Desmodium styracifolium*: (Family: Leguminosae; Common name: Osbeck)

Traditional Chinese medicine (TCM) has used the Chinese herb *Desmodium styracifolium* [111] extensively to treat urolithiasis with little to no negative effect [112]. When administered intravenously to dogs under anesthesia, preparations produced from the plant's dry leaves and stem enhanced coronary circulation reduced arterial BP, slowed heart rate, and reduced cardiac oxygen consumption [104]. It is investigated that the in vivo and in vitro cardiovascular pharmacology of *Desmodium styracifolium* [111]. The extract assisted in loosening methoxamine-pre-constricted isolated helical tail artery strips. *Desmodium styracifolium* extracts had a favorable chronotropic effect with no visible impact on contractile force. The bioassay is based on the measurement of ACE inhibition from the enzymatic cleavage of the substrate dansyltriglycine into diglycine and dansylglycine, which is labeled with a chromophore and a fluorescent dye [111]. Further research is required before considering it a primary treatment.

### *Lycopersicon esculentum*

#### *Lycopersicon esculentum*: (Family: Solanaceae; Common name: Tomato)

The tomato known as *Lycopersicon esculentum* L. is one of the most popular vegetables and the second-largest vegetable production in the world. It is an essential part of the "Mediterranean diet," which is closely linked to a lower risk of chronic degenerative diseases [113]. In people with mild, untreated HTN, a tomato extract called Lyc-O-Mato has been shown to reduce BP [114]. Treatment compliance was fairly high, and there were no documented negative effects of the medication [115]. As a result of tomato supplementation, OS is decreased and alterations in miRNA expression are induced. Additionally, the cross-sectional area decrease and improved diastolic function may be the result of these changes [116]. Tomatoes have elements that may help control BP. While not the only answer, including them in a balanced diet may improve cardiovascular health in general.

### *Pinus pinaster*

#### *Pinus pinaster*: (Family: Pinaceae; Common name: Maritime Pine)

Pycnogenol is a French maritime pine bark extract. It has been utilized all over the world as a food supplement, herbal medicine, and sustenance for many different degenerative conditions [117]. The most frequent conditions it is used to treat include vascular diseases and venous insufficiency. However, several diseases, including HTN, are being researched about it. Taking 200 mg of pycnogenol per day can help people with mild HTN decrease their BP. Blocking ACEs is thought to be the mechanism of action [118]. After 8 weeks of using Pycnogenol supplements, mildly hypertensive patients who did not need normal pharmacological medication therapy experienced a significant drop in SBP and serum thromboxane B2 levels [119]. For better cardiovascular health, beneficial integration of it could supplement current approaches.

### *Punica granatum*

#### *Punica granatum*: (Family: Lythraceae; Common name: Pomegranate)

Iran is the original home of the pomegranate that is known as *Punica granatum* L. The medical characteristics of this plant, in addition to its use as a fruit, have recently caught the attention of researchers from many different nations [120]. Pomegranate juice is gaining popularity as a fruit beverage. Studies show that pomegranate decreases ACE activity [121] by 37%. There have been a variety of outcomes from clinical studies. Following a year of daily consumption of 50 mL of pomegranate juice, an experiment found a lowering effect on SBP. Following 3 months of daily juice consumption of 240 mL, a different trial found no benefit [122]. Pomegranate can be used to help to regulate BP. Although it is not a sole treatment, using it in a healthy diet may help maintain cardiovascular health.

### *Raphanus sativus*

#### *Raphanus sativus*: (Family: Cruciferae; Common name: Radish)

Radish known as *Raphanus sativus* is a root vegetable that is grown and consumed all over the world and is regarded to be a staple of the human diet [123]. Atropine's inhibitory impact was lessened in tissues that had been administered it, demonstrating that it had a stimulating effect on the heart that was resistant to blocking by serotonin and adrenergic receptors. In the rat aorta with intact endothelium, it stopped atropine-blocking phenylephrine-induced contractions. The extract's safety in mice was demonstrated at concentrations of up to 10 g/kg. The plant's circulatory inhibitory effects can be used to treat HTN since they are mediated by muscarinic receptor activation [124]. Inhibiting lipid peroxidation, promoting liver and RBC catalase, and inhibiting XOD activities in animal tissues are all possible effects of the flavonoids (**12**) and vitamin C in radish [125]. The radish provides minerals that could be useful for lowering BP. Although their benefits are probably minor, they should be taken into account as part of a complete strategy for heart health.

### *Rhaptopetalum coriaceum*

#### *Rhaptopetalum coriaceum* Oliver: (Family: Scytopetalaceae)

An old method of treating HTN involves making a decoction of the plant's stem bark and soaking it in locally produced gin [126]. In addition to reducing noradrenaline and KCl-induced Ca^2+^ influx, this was achieved by inhibiting potential sensitive channels and receptor-operated channels. According to the in vitro data, nifedipine may not be as effective as the ethanol extract of *Rhaptopetalum coriaceum* as a calcium channel blocker [127]. It indicates that the endothelium is required for the relaxation response to the extract, which may be related to an intracellular rise of cyclic guanosine monophosphate (cGMP) [128]. The *Coriaceous graptopetalum* has unexplored possibilities for treating HTN. Traditional applications imply efficacy, although there is a lack of scientific support. Before considering its use, precaution and additional investigation are advised.

### *Solanum sisymbriifolium*

#### *Solanum sisymbriifolium*: (Family: Solanaceae; Common Name: Sticky Nightshade)

In traditional Paraguayan medicine, the perennial herb *Solanum sisymbriifolium* Lam. is used as a diuretic and an antihypertensive [129]. Traditional medicine in Paraguay uses *Solanum sisymbriifolium* a perennial herb with diuretic and antihypertensive effects. Rats with HTN conditions were used to test the crude hydroalcoholic root extract's hypotensive effects. BP significantly decreased after the extract was injected intravenously into rats with HTN. A dose-dependent hypotensive response was observed after the extract was administered orally to conscious hypertensive rats [130]. Nuatigenosido was identified as one of the extract's potentially active components in another investigation. The BP of Rats was lowered by nuatigenosido at doses of 100 g/kg and 1 mg/kg intravenously, while frog atrial myocytes' exceed amplitude was raised at 107 M. The outcomes suggested that nuatigenosido is crucial for the medicinal effects of the herb [131]. It can be argued that the clinical efficacy characteristics of this plant, such as hypotension, appear to be partially mediated by the vasodilatation and cardiotonic action generated by nuatigenosido [132]. The role of *Solanum sisymbriifolium* in the management of HTN. For managing BP, it is wise to seek advice from medical experts and use treatments.

### *Theobroma cacao*

#### *Theobroma cacao*: (Family: Malvaceae; Common names: Chocolate)

The rainforest regions of tropical America, from Mexico to Peru, are where cacao is native. The bark of cacao makes up around 84 percent of its composition, with the remaining percent consisting of seeds, pulp, and other ingredients [133]. Due to its high flavonoid (**12**) content, cocoa powder is used to help prevent CVD. Chocolate flavonoids (**12**) boost NO generation, improve vasodilation, and reduce endothelial dysfunction. An increasing amount of scientific evidence shows that eating 46 to 105 g of chocolate (*Theobroma cacao*) each day, with 214 to 400 mg of cocoa polyphenols, will reduce BP by roughly 5 mmHg and 3 mmHg, respectively [134]. The biological effects of cocoa polyphenols are garnering increasingly more attention. In fact, cocoa's high polyphenol concentration and extensive use in many foods make it particularly interesting from a nutritional and pharmacological perspective [135]. Flavonoids (**12**) found in *Theobroma cacao*, may have a beneficial effect on BP. However, due to its high-calorie content and other variables, it should only be used as a supplement, not as an individual treatment.

### *Uncaria rhynchophylla*

#### *Uncaria rhynchophylla*: (Family: Rubiaceae; Common name: Cat's Claw herb)

*Uncaria rhynchophylla* is one of the original plants used to make the significant Chinese crude medication Gou-teng, which is primarily used to treat brain disorders, HTN, convulsions, eclampsia, and epilepsy [136]. *Uncaria rhynchophylla* has been used in TCM to decrease BP and treat a variety of neurological conditions. The indole alkaloid hirsutine, which affects Ca^2+^ channels, is responsible for the hypotensive effect [137]. By affecting both the voltage-dependent Ca^2+^ channel and the Ca^2+^ storage, hirsutine is expected to lower intracellular Ca^2+^ levels [138]. Different from *Uncaria rhynchophylla* and its equivalent, a methanol extract of hooks from an Uncaria species demonstrated a potent and pervasive hypotensive effect in rats [139]. The extraction of the 3-indole alkaloid, glycoside, cadambine, dihydrocadambine, and isodihydrocadambine from the extract spurred more research. While the cadambine was demonstrated to be inert, the two were found to be the hypotensive principles [140]. Due to its effects on blood vessel relaxation, *Uncaria rhynchophylla* has potential effects as a therapy for HTN. Before considering it to be a reliable alternative, further thorough research is required.

### *Viscum album*

#### *Viscum album*: (Family: Santalaceae; Common name: Mistletoe)

A perennial, hemiparasitic shrub, epiphytic, called *Viscum album* that thrives on a variety of woody plant species, it is evergreen. It is a known pathogen, a medicinal plant, a native of Europe, and a mythological icon [141]. In the isolated and diffused heart model developed by Langendorff, aqueous leaves extracts of *Viscum album* significantly increased coronary vasodilator activity. The aqueous extract of *Viscum album* has some physiologically active elements that could operate as an inducer of the NO/soluble guanylate cyclase pathway [142]. The potential of extract to reduce BP was inhibited by propranolol. Atropine has no influence the extract's BP depression. The noradrenaline-blocking extract raised BP [118]. Mistletoe extract exerts an antihypertensive effect without changing HR, showing that sympathetic processes are involved [143]. Only one drawback is founded to *Viscum album* therapy, which needs to be confirmed by toxicology tests on cardiac muscle cell lines. This drawback is an elevated blood creatine phosphokinase myocardial band level [144]. Due to its vasodilatory effects, mistletoe has been studied for HTN. Before incorporating it into medical treatments, though, its effectiveness and safety must be thoroughly studied.

### *Zingiber officinale*

#### *Zingiber officinale*: (Family: Zingiberaceae; Common name: Ginger)

The spice ginger, also known as *Zingiber officinale*, is used in food and medicine throughout the world. The plant has several compounds that provide it with medical activities, including those that are anti-arthritic, antidiabetic, anti-fungal, and anti-cancer [145]. It improves blood circulation by relaxing the muscles that surround blood vessels. Anesthetized rats' arterial BP dropped dose-dependently (0.2–3.0 mg/kg) after being given a crude extract of ginger. It was determined that, like verapamil, Zo. Cr blocked Ca^2+^ channels by skewing the Ca^2+^ dose–response curves to the right. The inhibition of voltage-dependent calcium channels is thought to be the cause of ginger's BP-lowering effect [146]. To deoxycorticosterone acetate salt-induced hypertensive rats, pet ether extract (50 mg/kg/day; po), lowered BP, while PE (10 mg/kg/day; po) and KGE (30 mg/kg/day; po) to fructose-induced hyper. The mechanism of action could be aided by the serotonergic antagonistic feature [147]. Only a few human trials of ginger's hypotensive impact have been conducted, and most of them utilized a modest dose with unclear findings [148]. Ginger has hypotensive effects. Although it is not a solo treatment, adding ginger to a healthy diet may help manage HTN naturally.

### *Andrographis paniculata*

#### *Andrographis paniculata*: (Family: Acanthaceae; Common Name: Kalmegh)

*Astragalus paniculatus* in China, India, and other Southeast Asian nations, is a medicinal plant that has historically been used to cure colds, fevers, laryngitis, and several infectious disorders, from malaria to dysentery and diarrhea [149]. This is a traditional Chinese and Southeast Asian medicinal plant used to cure colds, fevers [150], upper respiratory and gastrointestinal tract infections, hepatitis, herpes, and CVD [76]. ACE, β-adrenoceptors, and autonomic ganglion receptors are all inhibited by andrographispaniculate [121, 151]. Its extracts contain a number of diterpenoid chemicals, including 14-deoxy-11,12-didehydroandrographolide, andrographolide (**02**), and 14-deoxyandrographolide (**03**) [152], which have anti-inflammatory, antibacterial [153], antioxidant, and hypotensive properties. Its chloroform extract can activate NO synthesis and, as a result, promote NO production in endothelial cells, causing smooth muscle relaxation by blocking Ach action [154]. Through lowering ROS and ACE activity, *Astragalus paniculatus* lowers BP in impulsively hypertensive rats [76]. According to Awang et al. [152] In isolated rat hearts, 14-deoxy-11,12-didehydroandrographolide lowered vascular resistance. The researchers found that a crude extract containing a significant amount of 14-deoxy-11,12-didehydroandrographolide significantly lowers BP because it increases NO release, which causes vasodilation. Additionally, 14-deoxy-11,12-didehydroandrographolide inhibits voltage-gated Ca^2+^ channels, lowering Ca^2+^ concentrations inside the cell. Endothelial protection and smooth muscle relaxation were achieved as a result of the L-type Ca^2+^ current and high K^+^ activation pathways being inhibited by the *Andrographis paniculata* chloroform extract. ACE inhibitory activity, β-adrenoceptors, and autonomic ganglion receptors are thought to be the mechanisms through which diterpenoid lactones as DDA from *Andrographis paniculata* exert their hypotensive effects [75, 155–157]. The ability of *Andrographis paniculata* to relax blood arteries makes it a potential treatment for treating HTN. Before deciding to use it as a primary treatment, additional reliable clinical data is required.

### *Bidens pilosa*

#### *Bidens pilosa* L.: (Family Asteraceae; Common Names: Broom Stick)

An annual herb with a global reputation for being used to treat several diseases, *Bidens pilosa* L. has been extensively researched for the biological activity of its extracts, fractions, and isolated components [158]. The bioactive components have been used to treat bacterial, cancer, obesity, HTN, malaria, and CVD [43, 159], making it a popular plant today. There are at least 60 flavonoids (**12**) in *Bidens pilosa*, and they contains a range of chemical components [159]. As a result, preparations of this plant are extensively used as medicine to treat around 40 different diseases [159, 160] via a variety of anticipated [161] processes, including vasodilation, lipid profile improvement, free radical scavenging, insulin-sensibility, calcium channel blocker, and so on [73, 162–164]. Quercetin (08) improves endothelial function by boosting NO production and/or bioavailability. It has been confirmed quercetin's (08) potential to lower and prevent BP [159, 161, 162]. High doses of *Bidens pilosaca* leaf extracts lower plasma creatinine levels, which enhance plasma cholesterol levels. As a result, they theorized that *Bidens pilosa*'s hypotensive action is independent of insulin sensitivity [165]. In multiple normotensive and hypertensive rat models (caused by fructose), the aqueous and CH_3_Cl leaf extracts of *Bidens pilosa* can reduce and prevent high BP during the course of a 3-week continuous therapy [161, 166]. Dimo et al. and Bartolome et al. [159, 162] have demonstrated the vasorelaxant effects of *Bidens pilosaca*, and Nguelefack et al. have seen increasing levels of a neutral to induce relaxation in noradrenaline and potassium chloride pre-constricted rat aortas [167]. Their theory was that calcium channel antagonism or a cyclooxygenase metabolite produced vasodilation [155]. They went on to assert that the vasodilation process had nothing to do with the ATP-dependent K^+^ channel [159, 167]. Due to its diuretic characteristics, the traditional treatment of *Bidens pilosa* may help with HTN. Before considering its use for BP control, it is imperative to consult with healthcare professionals.

### *Camellia sinensis*

#### *Camellia sinensis*: (Family: Theaceae; Common Name: Tea)

One of the oldest and most widely favored medicinal drinks consumed worldwide is green tea. The leaf of the "*Camellia sinensis*" plant is used to make this product [168]. Catechins (**09**) are the most common flavonoids (**12**) found in tea (−)-epicatechin-3-gallate (ECG), (−)-epicatechin (EC), (−)-epigallocatechin-3-gallate (EGCG) (**04**), (−)-epigallocatechin (**05**) (EGC, primary component) [169]. As a result of an enzymatic process, the catechins (**09**) are transformed into flavins, and the origins are known to be effective vasodilators. Due to enhanced NO release and a decrease in OS and dimethylarginine levels, catechins (**09**) are also responsible for a considerable improvement in blood flow [170]. Anti-diabetic, anti-inflammatory, antibacterial, antihypertensive, and anti-cancer activities have been found in *Camellia sinensis* aqueous extract [171]. People who drank green and black tea regularly had a lower risk of HTN [169]. Based on a meta-analysis, the regular tea drinking had a substantial decreasing effect on DBP [172], however other studies found that it is concentration dependent [50]. Drinking concentrated, only green tea regularly can lessen the risk of CVD mortality [48]. Daily ingestion of black tea extract for seven days decreases SBP because the o-methylated EGCG content inhibits the ACE. After drinking lyophilized green tea extract, mildly hypertensive patients' SBP (4.9 mmHg) and DBP (4.7 mmHg) decreased significantly [173, 174]. Green tea can lower BP in a variety of ways, including maintaining a balance of vasoconstricting, vasodilating, and hyperpolarizing hormones [172]. It increases NO production by stimulating antioxidant enzymes and inhibiting pro-oxidant enzymes, which helps to improve heart function and regulate ROS production [175]. Studies have shown that medicinal plants can treat HTN using a variety of mechanisms, including vasodilation, NO production, inhibition of the renin-angiotensin system, activation of intracellular cGMP, and expansion of the vasodilator effect [176]. Tea made from *Camellia sinensis* contains substances that may only slightly help with BP control. Moderate tea consumption may support a healthy lifestyle for cardiovascular health.

### *Crataegus* spp.

#### *Crataegus* spp.: (Family: Rose; Common Name: Hawthorns)

For many years, different cultures have used the medicinal benefits of hawthorn (*Crataegus* spp., a genus with about 300 species) for a range of therapeutic purposes [177]. Hawthorn plants have been used in traditional medicine to treat CVDs for a long time. Hawthorn medicine (500 mg daily for 10 weeks) can lower DBP in hypertensive patients [178]. A considerable BP decrease occurs only after the medication is given in higher doses over a longer period [179]. These plants also include oligomeric proanthocyanidins such as procyanidin in, procyanidin B-2, and flavonoids (12) like vitexin, rutin, and others. Endothelial cells and VSMCs respond well to hawthorn extracts [180]. The extract reduces the levels of VCAM-1, IL-6, NF-κB, and iNOS, all of which are pro-inflammatory [86, 181]. More in vivo, in vitro, and clinical experiments are needed to investigate the connection between the chemical composition of these plants, particularly hawthorn, and their modes of action in the treatment of different disorders [88, 182]. Hawthorn has potential in the treatment of HTN through promoting heart health and vasodilation. To improve standard treatments, it should be used carefully and combined with medical advice.

### *Cymbopogon citrates*

#### *Cymbopogon citrates*: (Family: Gramineae; Common Name: Lemongrass)

*Cymbopogon citratus*, also known as lemongrass, is a plant that is frequently used in folk medicine for a variety of diseases. Yet further research is needed to fully understand its anti-hypertensive potential [63]. Researchers have found a variety of compounds in the herb's leaves and stems, including flavonoids (12), alkaloids, essential oil, tannins, phenols, saponins, and anthraquinones [183]. Citral, the major ingredient in *Cymbopogon citratus*, has antibacterial, antioxidant, chemoprotective, and antispasmodic properties when used alone or in combination with other ingredients. Citral prevents L-NAME from attenuating the generation and release of NO, resulting in vasorelaxation. Furthermore, the extract from the leaves may affect prostacyclin production, resulting in relaxation. [184]. In rats, *Cymbopogon citratus* fresh leaf extract, when combined with other herbal treatments like *Citrus medica* fruits extract and *Persea americana* fresh leaf extract, can reduce HTN caused by sugar and ethanol [63]. Ray discovered that twice-daily therapy with a lemongrass decoction reduced mean arterial pressure significantly [185]. Lemongrass oil can prevent ROS from forming. Citral suppresses iNOS and NF-κB activity, as well as having anti-inflammatory characteristics [72]. To increase the development of new herbal medicines and study their therapeutic potential, attention to medicinal plants has recently been focused on pharmacological research and phytochemical screening of secondary metabolites [186]. *Cymbopogon citratus* has a long history of supporting BP control. Because of its potential for minor effects, it can be used in addition to more well-known HTN control techniques.

### *Nigella sativa*

#### *Nigella sativa*: (Family: Ranunculaceae; Common Name: Black Cumin)

Asthma, diarrhea, and dyslipidemia are just a few of the diseases that are treated and prevented with the seeds of *Nigella sativa* throughout the world in folk (herbal) medicine [187]. When *Nigella sativa* is ingested, BP is reduced [188, 189]. Most of the positive effects of seeds are due to thymoquinone, a major active component in black seed essential oil [190]. Another active component of *Nigella sativa*, thymol (**16**), has been demonstrated to reduce BP in endothelium cell membranes via an endothelial-independent mechanism (inhibition of calcium ion influx through calcium channels), followed by vasorelaxation [189]. The other bioactive elements include proteins, fatty acids, alkaloids, antioxidants, essential oils, flavonoids (**12**), and antioxidants. In people with mild HTN, Wong found that taking an *Nigella sativa* extract twice a day for 8 weeks dramatically lowered BP [191]. *Nigella sativa* may be effective in the treatment of HTN because it reduces ROS [192]. The oil of *Nigella sativa* can lower both DBP and SBP [193]. According to Ahmad et al. [71], TQ (Thymoquinone) induces vasodilation by lowering the production and release of COX-1 and COX-2 metabolites. Black cumin functions as an anti-inflammatory agent by reducing NF-κB [155, 194]. There is evidence to support including *Nigella sativa* and its bioactive ingredients in your diet regularly to improve your health [71]. The antioxidant and vasodilatory properties of the *Nigella sativa* indicate a potential for HTN. Its function should be considered supplemental, and more research is required before making clear suggestions.

### *Panax ginseng*

#### *Panax*: (*Panax ginseng*, Panaxquinquefolius; family: Araliaceae; Common Name: Japanese Ginseng, Asian or Korean Ginseng)

*Panax ginseng* which is native to Korea and China and is used as an herbal treatment [195]. For centuries, the Panax ("all healer") was thought to be capable of treating all human diseases [196]. Panax roots, in both solid and liquid form, have long been used in folk medicine for a variety of therapeutic and pharmacological purposes [197]. The biological effects of this medicinal plant include a low BP antidiabetic, anticancer, antioxidation, vasorelaxation, anti-carcinogenic, anti-allergic, anti-inflammatory, and more [196, 198]. Although it is generally known that ginseng reduces BP, it is claimed that it can also raise BP to regulate hypotensive circumstances, most likely by altering vascular features, changing the autonomic nervous system, or adapting the baroreflex of arteries [199]. In both patients with moderate HTN and healthy people, it is discovered that ginsenoside from *Panax ginseng* has a substantially decreasing effect on SBP and DBP [200]. Ginsenoside Rg3 has a stronger effect on eNOS expression, which leads to more NO production and vasorelaxation [200]. Ginseng also inhibits the release of catecholamines from the adrenal glands, lowering BP [197].  *Panax ginseng* shows promise in the therapy of HTN. Although it is not a stand-alone treatment, its restricted use may support existing cardiovascular health treatments.

### *Salvia miltiorrhizae*

#### *Salvia miltiorrhizae*: (Family: Labiatae; Common Name: Danshen)

TCM uses *Salvia miltiorrhiza*, also known as Danshen in Chinese. It is a *Salvian perennial* flowering plant that has long been valued for its use in TCM [90]. Root extracts have properties that are antimicrobial, and antioxidant, and that prevent CVD and inflammation [90, 91]. Danshen's roots extract reduces heart rate and SBP by boosting eNOS signaling synthesis and magnifying NO production to encourage vasodilation [201]. Tanshinone IIA causes vasodilation without the need for endothelial cells to be involved [201]. The metabolite of Danshen increases intracellular Ca^2+^ influx through altering receptor and voltage-dependent calcium channels [202]. Danshen also inhibits ACEs, lowering BP [203, 204] which has been connected to antihypertensive effects in the lab [205–208]. It is hypothesized that RA and RS can successfully suppress Ang II expression, stop RAS activation, inhibit TGF-1 expression, postpone the progression of the RIF, and protect against HTN and damage to cardiac function [209]. The cardiovascular benefits of *Salvia miltiorrhizae* suggest that it may be useful in the treatment of HTN. Consideration might be given to incorporating it with medical advice to improve overall BP control.

### *Rauvolfia serpentina*

#### *Rauvolfia serpentina*: (Family: Apocynaceae; Common Name: Devil Pepper)

For many years, the root of a flowering plant belonging to the Apocynaceae family called Sarpgandha has been utilized in Ayurveda to cure several diseases that at first glance seem to have little in common with one another [210]. Rauwolfia Treatment of HTN is the most common application of serpentine (**10**). It slows down nervous system activity, slows down heartbeat, and opens up blood vessels. Rauwolfia also includes steroids, flavonoids (**12**), and tannins in addition to fatty acids, alcohols, sugars, and glycosides. All parts of the plant contain indole alkaloids, but the root bark contains higher concentration. Ajmalinine, ajmalidine, ajmaline, and raubasine, papaverine are a few of the indole derivatives that have been identified [211]. The most popular indole derivative, reserpine (**11**), has antihypertensive properties and lowers both SBP and DBP [210–213]. Storage vesicles in the cell become irreversibly attached to VMAT2 and 'leak' their contents into the cytosol, including monoamine, where MAO-A enzymes then contaminate the cytosol. Monoamine modification is age-independent [214]. The entire topic of *Rauvolfia serpentine* therapy for HTN has been studied up to this point, including discussions on the history of the plant, its various species and types, nomenclature, geographic distribution, chemistry, pharmacologic actions, and clinical studies, reported on the subject from all over the world [8, 215–217]. In the past, *Rauvolfia serpentine* was used to treat HTN. BP is lowered by its primary ingredient, reserpine (**11**), although side effects and current alternatives necessitate medical supervision and careful evaluation.

### Stevioside

The leaves *Stevia rebaudiana* (Bertoni) are used to make the natural sweetener stevioside. The literature on Stevia is discussed, as well as the presence of its sweeteners, their biosynthetic process, and any toxicological implications [218]. In both animal and human experiments, it has been shown to reduce BP. Intravenous doses of 50, 100, and 200 mg/kg produced dose-dependent hypotensive effects on both SAP and DAP in awake SHRs. The most significant reductions in SAP and DAP were 31.4 to 4.2 percent and 40.8 to 5.6 percent, respectively [219]. Crude stevioside at doses up to 15.0 mgkg1 d1 had no antihypertensive effect in previously untreated mild HTN patients [220]. Stevioside inhibits extracellular Ca^2+^ influx and the generation of a vasodilator prostaglandin, which may alter vascular resistance. Stevioside also causes diuresis and natriuresis, resulting in a decrease in extracellular fluid volume [221]. This work demonstrated the efficacy of stevioside as a natural antihypertensive agent and suggested that its hypotensive action may be caused by a Ca^2+^ influx inhibition mechanism [221]. Due to its possible vasodilation, stevioside may have a minor effect on BP. However, its efficacy in managing HTN is still limited, necessitating additional study and medical advice.

### Orientin

Water-soluble flavonoids (**12**) include orientin (**15**). With two ether groups and one ketone group, orientin (**15**)'s chemical structure reveals that phenol groups make up the majority of its composition [222]. *Phyllostachys nigra* bamboo leaves, which have been used in Chinese medicine, are the source of the substance known as "orientin (**15**)." Additionally, orientin (**15**) inhibits vasoconstriction caused by norepinephrine, CaCl_2_, and KCl in a concentration-dependent, non-competitive manner as well as contractions brought on by phenylephrine during both the initial fast release and sustained phases. Orientin (**15**) can encourage endothelial cells to generate NO [223, 224]. The polyphenolic chemicals found in BS were therefore the source of the ACE inhibitory actions of the methanol extracts of BS [225]. Through its antioxidant and vasodilatory properties, the flavonoid (**12**) orientin (**15**), which is present in many plants, has the potential to treat HTN. Even though it appears promising, additional research is required to determine its effectiveness and safety in the treatment of HTN. For advice, always seek the advice of a healthcare professional.

### Ostruthol

Natural products have been utilized as part of herbal treatments for millennia to alleviate and treat a variety of diseases. Their unusual chemical diversity provides a wide source of drug-like compounds with a variety of biological actions, accounting for their substantial contributions to drug development [226]. Its activity against K^+^-spasms is much stronger than its activity against norepinephrine contractions [227]. However, more research is necessary before recommending it due to its clinical significance and safety. It might be an additional choice for managing HTN, but consulting with a healthcare professional is important.

### Flavonoids

Numerous animal studies conducted over the past 10 years have demonstrated the flavonoids' (**12**) positive effects on the cardiovascular system. The anti-HTN actions of flavonoids (**12**) or extracts of plants rich in flavonoids (**12**), as well as their potential mechanisms, were therefore discussed. Flavonoids (**12**) have been identified to have a variety of potential effects on BP. The clinical use of flavonoids (**12**) as an adjuvant or core drug is not supported by the extensive evidence of their potential in the treatment of HTN, in addition. Therefore, the discussion also takes into account the synergistic effects of flavonoids (**12**) with other medications, pharmacokinetic research, clinical trials, and the safety of flavonoids (**12**) [228]. Among the many bioactive polyphenolic chemicals found in dietary plants and herbs, flavonoids (**12**) are a varied group. Flavonoids (**12**) have cardio-vasculoprotective properties that can delay or stop the progression of several CVDs, including HTN. Anthocyanin consumption and risk of myocardial infarction, flavanone intake and risk of ischemic stroke, and flavanols intake and risk of type 2 diabetes mellitus. Human randomized controlled trials (RCTs) demonstrate that quercetin (**08**) and catechins (**09**) significantly decrease BP. By enhancing NO bioavailability, lowering endothelial cell OS, or altering vascular ion channel function, flavonoids (**12**) exert their antihypertensive effects of antihypertensives and potential ways through which they control BP. The security of these substances as well as their prospective application in the control and treatment of HTN [229]. Due to their antioxidant and vasodilatory effects, flavonoids (**12**), which are widely present in fruits and vegetables, show potential in the control of HTN. Their inclusion in diets may improve cardiovascular health.

### Polyphenol

Dietary polyphenols affect BP, HTN, and vascular health. There is proof that several foods high in polyphenols, such as berry fruits with high anthocyanin content, cocoa and green tea with high flavan-3-ol (**17**) content, almonds and pistachios with a high hydroxycinnamic acid content, and soy products with high isoflavone (**13**) content, can lower BP [230]. The NO-cGMP pathway and ACE inhibition are involved in the antihypertensive phytotherapy of polyphenol-rich diets for defense and enhancing endothelial function with arterial relaxation. OPCs trigger a laminar shear stress response in endothelial cells, induce endothelium-dependent vasodilation, reduce the synthesis of the vasoconstrictor ET-1, and also block the activities of metalloproteinases such as ACE, hence reducing BP [231]. Polyphenols, which are present in foods like berries and tea, show promise in the treatment of HTN. They may improve overall cardiovascular health when included in diets.

### Alkaloids

*Uncaria rhynchophylla* alkaloid has been demonstrated to have both a substantial protective impact on human umbilical vein endothelial cells (HUVECs) and an antihypertensive effect. Further research has shown that *Uncaria rhynchophylla* alkaloid reduces the inflammatory response in artery walls, hence inhibiting the release of intercellular adhesion molecule-1 (ICAM-1) and vascular cell adhesion molecule-1 (VCAM-1) [232, 233]. The design of selective inhibitors may be studied using phytopharmaceuticals, such as alkaloids, which have a rich medicinal history and diverse clinical applications. The study came across the alkaloids that have already been put to the test and their significant outcomes, which prompted additional research on these and many other alkaloids for the identification of new efficient and selective ACE inhibitors [234]. It is used to treat HTN. The significance of medical guidance and modern therapies is highlighted by current alternatives and probable adverse effects.

### Lycopene

Indirectly increasing NO production in the endothelium, lycopene (**14**) decreases OS, acts as an antioxidant, and lowers BP. In 54 patients with moderate HTN who were already taking ACE inhibitors or calcium channel blockers after 6 weeks of tomato extract supplementation, there was a significant decrease in both SBP and DBP, indicating a role for lycopene (**14**) in the treatment of HTN [235]. Lycopene (**14**) supplementation was observed to lower SBP in prehypertensive and hypertensive patients in a meta-analysis, but it did not affect DBP [236, 237]. Lycopene (**14**), which is rich in tomatoes and has antioxidant effects, may help with BP control. While not a cure-all, consuming lycopene (**14**)-rich foods may improve cardiovascular health.

### Cinnamon

Cinnamon (*Cinnamomum Zeylanicum*) has promising antihypertensive activities and may even increase the effectiveness of amlodipine in lowering BP. SBP was significantly reduced to an average of 145.57 2.6 mmHg as a result of co-administration with amlodipine, a drop of 15.48% when compared to the hypertensive control group. Cinnamon consumption significantly lowers BP levels in hypertensive rats and improves the PK characteristics of amlodipine [238]. Although it is used in food preparation for flavor and taste, cinnamon may also play a part in the regulation of BP and glucose metabolism. Therefore, adding helpful dietary ingredients, including cinnamon, that have favorable effects, in addition to lowering the number of ones that have negative effects on BP and insulin function, may help regulate BP [239]. Cinnamon has a limited ability to reduce BP. While adding it to a healthy diet may have some advantages, established therapy and medical supervision are necessary for complete HTN management.

### Garlic

Garlic (*Allium sativum*) and several of its bioactive components may have an antihypertensive effect. With a particular focus on S-allyl cysteine and allicin (**01**), the review's objective is to give an in-depth discussion about the molecular, biochemical, and cellular rationale underlying the antihypertensive characteristics of garlic and its bioactive ingredients. The function of S-allyl cysteine and allicin (**01**), two garlic bioactive, in modifying several parameters thought to be involved in the development of HTN. OS, NO bioavailability, hydrogen sulfide generation, angiotensin-converting enzyme activity, NF-κB expression, and vascular smooth muscle cell proliferation are some of these markers [240]. By boosting NO production [241], inducing vasodilatation with hydrogen sulfide [242], and inhibiting angiotensin-converting enzyme activity, garlic extracts have been found to have anti-hypertensive actions. Garlic extract is a vasorelaxant and may lessen the atherogenic potentials of cholesterol in rats [243]. The essential vasodilatory and antioxidant effects of garlic point to possible applications in BP control. Garlic in the diet could successfully support current HTN treatments even though it is not a cure-all.

### Omega-3 fatty acids

The evidence for the benefits of omega-3 PUFA on BP is inconsistent, and the reduction shown in many trials is moderate. However, older people and hypertensive participants showed greater reductions when supplementing with doses of up to 3 g/day of fish oil or blue fish. However, in hypertensive people, consuming omega-3 can lower cardiovascular risk by decreasing the rise in BP. Therefore, more clinical research is required to determine how supplementing with -3 PUFA reduces cardiovascular risk in a homogeneous population [244]. Impaired vasodilation involving the malfunctioning of several vasodilatory pathways is a common feature of HTN. BP and vasodilation can be lowered by PUFAs with an omega-3 chain [245]. Fish and dietary supplements containing omega-3 fatty acids have been shown to have cardiovascular advantages, including the potential to lower BP. Along with medical advice, including them in diets can help manage HTN.

### Linalool

A common remedy for HTN, linalool (**06**) is a monoterpene alcohol found in various aromatic medicinal plants native to Brazil [246]. The function of linalool (**06**) and its underlying mechanism in VSMC physiology. After treating VSMCs with angiotensin II (Ang II), MTT and western blotting tests were used to determine whether linalool (**06**) had any impact on the induced proliferation and migration of VSMCs [247]. A natural substance called linalool that is present in several plants may be useful in treating HTN. It may aid in relaxing blood vessels and lowering BP. Before recommending it as a primary therapeutic choice, more research is required.

### NG-nitro-l-arginine methyl ester

Investigations were conducted to determine the impact of an aqueous *Mentha cordifolia* extract on the oxidative status in NG-nitro-l-arginine methyl ester (**07**)-induced HTN [248]. The antihypertensive impact of *Phragmanthera incana* leaf ethanol extract in rats with HTN brought on by NG-nitro-l-arginine methyl ester (**07**) [249]. In the treatment of HTN, NG-Nitro-l-Arginine Methyl Ester may be crucial. Because it inhibits NO synthase, it aids in BP control. However, due to possible side effects, its clinical use should be done with care.

## Antihypertensive seafood compounds

The seafood compounds used to treat HTN because of having antihypertensive activity.

### Phlorotannins

**Fig. 8:**
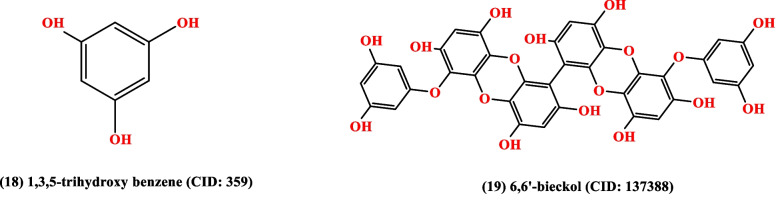
The chemical structures of seafood compounds. These compounds have antihypertensive activity

"Phlorotannin" (1,3,5-trihydroxy benzene) (**18**) is a class of polyphenolic chemicals found in brown algae, accounting for around 5–12 percent of the dry mass [250]. They are commonly found in brown seaweeds, particularly the Ecklonia species, and offer a variety of advantageous biological properties, including anticancer, antiallergic, and antihypertensive benefits [250–253]. Given their prior discoveries, it is not surprising that phlorotannins are connected to the ACE I inhibitory activity postulated by Olivares-Molina and Fernández [254]. According to reports, Dieckol is a non-competitive ACE I inhibitor that induces NO generation in EAhy926 cells without having any cytotoxic effects. However, its inhibitory action is not equivalent to captopril's [255]. Although the alleged low IC_50_ appears to support this, the related standard deviation shouldn't be far larger than the IC_50_ mean value [256]. *Ecklonia cava* was the source of the 6,6′-bieckol (**19**) (Fig. 8). With an IC_50_ Kjellman is less active than phlorofucofuroeckol A at inhibiting the ACE enzyme. In addition, Ko et al. [257]. 6,6′-bieckol (**19**) may be effective in the management of HTN. It appears that a dibenzo-1,4-dioxin moiety is necessary for ACE I inhibition, even though the structure–activity connections for phlorotannins are still being worked out [256]. Phlorotannins are hypothesized to inhibit ACE I because of their capacity to bind to proteins and the consequent reduction in ACE I binding effectiveness [255]. Phlorotannin's ability to bind to proteins is determined by its length and structure; phloroglucinol pentamers or hexamers are clearly superior inhibitors [258, 259]. These approaches have mostly been employed for separation monitoring and qualitative profiles, with few data on quantification or quality control development reported [260]. Phlorotannins from some seaweeds have the potential to lower BP by altering the activity of BP-regulating enzymes. To determine their clinical value and safety, more research is needed.

### Polysaccharides

Polysaccharides are natural resources for vitamins and medications, which have gained popularity in recent years. Natural polysaccharides have been proven to have less adverse effects, but their bioactivities were challenging to compare to those of manufactured medicines due to their inherent physicochemical features [261]. Endothelin1 is the main constituent of the endothelin system, interconnects with two types of endothelin receptors inside cells. Major CVDs have been demonstrated to be candidates for endothelin receptor inhibition, particularly of the ETA subtype [262–264]. *Pelvetia canaliculata* (Linnaeus) Decaisne & Thuret, a brown alga, can be used to make d-polymannuronic sulphate, a chemical of the carbohydrate type that has been shown in vivo to have both acute and preventative hypotensive activity. d-Polymannuronic sulphate had a therapeutic potency of 50 mg/kg, which was on par with captopril's (14 mg/kg) level [265]. Polysaccharides are classified as specialty chemicals rather than commodity chemicals, including both natural gums and their derivatives. They are used in industrial applications as thickening and sizing agents [266]. Seafood, such as seaweed, contains antihypertensive polysaccharides that may lower BP in a number of different ways. Before making a clinical recommendation, more research must be done on their therapeutic potential.

### ACE inhibitory peptides

ACE [121] is a major enzyme in the renin-angiotensin system (RAS) that converts angiotensin (Ang) I into angiotensin II (Ang II), a vasoconstrictor that causes high BP [267]. Patients with HTN around the world are treated with ACE inhibitors. Synthetic chemical drugs that are frequently used in the treatment and prevention of HTN include captopril, enalapril, alacepril, and lisinopril [121, 268]. Peptides made from partially hydrolyzed food proteins by enzymes have drawn a lot of attention recently. Bioactive peptides with a wide range of health advantages have been created by food protein hydrolysates and have all been categorized and reported. These peptides can be produced during enzymatic digestion or food processing even if they are inactive inside the original protein sequence [121]. Natural source ACE [121] inhibitors improve the chances of dietary control of HTN. The structure of some sequences substantiated the presence of peptides with ACE-inhibitory, antioxidant and immunomodulatory activities [269]. By blocking the enzymes that control BP, ACE inhibitory peptides found in seafood have the potential to reduce BP. Their effectiveness and safety call for further scientific investigation for clinical use.

### Peptides from marine fish

Using the proper proteolytic enzymes, bioactive peptides can be isolated from the enzymatic hydrolysis of various marine fish sources. To develop bioactive peptides, marine fish products can be hydrolyzed using proteolytic enzymes obtained from plants, animals, and microorganisms [270]. Lineweaver–Burk plots have been used to kinetically assess the competitiveness of several antihypertensive peptides against ACE activity [271]. Antihypertensive peptides typically have a different mechanism of action than synthesized medicines. ACE inhibitory peptides interact very differently from synthesized medicines because they actively compete with ACE instead of generally blocking it. Angiotensin I is converted into angiotensin II by ACE by cleaving off a brief peptide. Instead of fighting angiotensin I, ACE interacts with antihypertensive peptides. Antihypertensive peptides work by suppressing the production of angiotensin II to relax artery walls and decrease fluid volume. Antihypertensive peptides hence boost blood and oxygen flow to the heart, liver, and kidneys while also improving cardiac function [272]. Because they block BP-regulating enzymes, peptides from marine fish have the potential to be used as natural antihypertensive medications. For safe and efficient integration, their role necessitates thorough scientific evaluation.

### Seaweed polyphenols

Bioactive peptides and protein hydrolysates from seaweeds have been shown in numerous studies to have antihypertensive effects [273], as those from, among others, *Undaria pinnatifida* [274], *Palmaria palmata* [275], *Porphyra columbina* [276], and *Porphyra yezoensis* [277]. The antihypertensive impact associated with these algae may also be related to other bioactive substances found in seaweeds. This might apply to polyphenols. Similar to the antihypertensive action previously discovered for other phenolic derivatives from land plants, such as flavonoids (**12**), these polyphenolic chemicals' capacity to exert antioxidant effects can also be responsible for the indicated antihypertensive activity [278, 279]. In contrast, polyphenols have been reported to function as ACE inhibitors [251, 255]. Angiotensin II, a strong vasoconstrictor involved in the etiology of HTN, is produced when angiotensin I is converted to ACE, a zinc-containing protease. ACE also degrades the vasodilator bradykinin. This enzyme is essential for the regulation of BP. As a result, its inhibition has emerged as a primary focus for managing HTN. This explains the development of various ACE inhibitors, which are frequently regarded as one of the primary therapeutic approaches for CVDs in people [280]. Seaweed polyphenols could be showed potential in the treatment of HTN. Additional study is required to determine their therapeutic value and safety.

## Conclusion and future direction

Traditional medicine refers to a broad range of centuries-old, culturally specific health care practices that were in use before the introduction of science to medical issues. In the end, throughout the past few decades, there has been a rise in the prevalence of HTN worldwide. The needs of the clinic are still obscure despite the use of numerous antihypertensive medications with various mechanisms of action [281]. The development of new lead compounds for the treatment of HTN has relied heavily on natural products and will continue to do so. To identify potential leads for structural alterations and optimization to produce future antihypertensive medications that are more effective and secure, numerous herbal treatments for HTN and plant extracts still need to be researched and identified [223]. The plants that are most frequently used to manage and treat HTN as well as their modes of action. Several factors, including endothelial function, ROS production, pro-inflammatory signaling, platelet activation, opening and closing of various ion channels, ACE inhibition, gene expression, and other pharmacological activities of natural plants and their isolates, influence the pathogenesis of HTN. According to the findings of relevant clinical and experimental research, herbal medicines will surely gather more attention in the future due to their wide spectrum of efficacy [7]. With a focus on the underlying molecular mechanisms of action within plants, herbs, phytochemicals, and marine components, this review has provided a thorough exploration of contemporary therapeutic approaches for treating HTN. Natural substances have significant potential as complementary therapies to established antihypertensive treatments, as they combine conventional knowledge with modern scientific findings. The complicated relationship of bioactive compounds from different sources indicates their ability to alter important pathways that control vascular function, OS, and BP. The evidence provided implies that these drugs have the potential for enhancing the available therapy options for HTN. Further research is required because of issues with standardization, bioavailability, and the transition from the bench to the bedside. Natural chemicals provide numerous benefits, including their potential synergistic effects, which can be utilized for future drug development and personalized therapy strategies. This research highlights the need for continuous multidisciplinary partnerships between traditional medicine practitioners, pharmacologists, and physicians to address the increasing global prevalence of HTN. We are in a position to begin a new era of comprehensive and efficient solutions for treating HTN and ultimately lowering the associated cardiovascular morbidity and mortality by utilizing the power of molecular pathways present in plants, and seafood components.

## Data Availability

No new data was generated for this review.
